# Co-detection and genomic characterization of avian rotavirus A, avian orthoreovirus, and chicken megrivirus-C using nontargeted metagenomic surveillance in Indian broiler chickens

**DOI:** 10.3389/fcimb.2026.1690222

**Published:** 2026-02-27

**Authors:** Henry M. Kariithi, Jeremy D. Volkening, Sarah N. Mueni, Mohamed A. Helmy, Claudio L. Afonso, Pushparaj P. Chaudhari, Eduardo L. Decanini

**Affiliations:** 1 Biotechnology Research Institute, Kenya Agricultural and Livestock Research Organization, Kikuyu, Kenya; 2 BASE_2_BIO, Oshkosh, WI, United States; 3 Department of Biochemistry, Jomo Kenyatta University of Agriculture and Technology, Nairobi, Kenya; 4 Boehringer Ingelheim Middle East and North Africa, Dubai, United Arab Emirates; 5 Boehringer Ingelheim India Pvt. Ltd., Mumbai, Vikhroli East, Maharashtra, India

**Keywords:** coinfections, enteric virome, metagenomic surveillance, ntNGS, poultry health, reassortment, IMETA (India, Middle East, Turkey, and Africa) region

## Abstract

Nontargeted metagenomic surveillance of the poultry enteric virome reveals underrecognized threats to poultry health and productivity in intensive production systems. In South Asia, avian rotavirus A (AvRV-A) and avian orthoreovirus (ARV) are frequently detected in broilers by conventional diagnostics, whereas chicken megrivirus genotype C (ChMeV-C) is often identified through metagenomic surveillance. Often present in both clinical disease and coinfections, these viruses may impair gut function, immune responses, and growth performance, yet their genomic diversity and evolutionary dynamics in poultry remain poorly characterized. Here, we report complete genomes of AvRV-A, ARV, and ChMeV-C strains co-detected via nontargeted metagenomic next-generation sequencing (ntNGS) in a pooled cloacal sample comprising 150 commercial broiler chickens (19 and 33 days old) collected from three commercial farms in Kamrup Rural District, Assam, Northeast India. Despite routine vaccination, all three flocks experienced > 10% mortality, poor weight gain, and postmortem lesions including pale kidneys and hepatomegaly. Phylogenetic analyses revealed segmental clustering in ARV and AvRV-A consistent with reassortment-driven divergence, though not supported by detectable recombination, while ChMeV-C clustered within a distinct C1 sublineage, suggesting intercontinental lineage connectivity and highlighting the need to expand regional genomic baseline data. We also identified nonsynonymous single nucleotide polymorphisms in several key viral proteins, including RNA-dependent RNA polymerases (VP1 of AvRV-A, λB of ARV, and 3D of ChMeV-C), capsid proteins (VP2 and VP7 of AvRV-A, λA and σB of ARV, and VP0 and VP1 of ChMeV-C), and replication-associated nonstructural proteins. These findings expand the genomic baseline for poultry enteric viruses in South Asia, reveal novel polymorphic signatures, and underscore the value of ntNGS-based metagenomic surveillance in virus detection, diversity monitoring, and informing vaccine and biosecurity strategies.

## 1 Introduction

The enteric virome of poultry includes a range of viruses that, often in subclinical form or coinfections, pose challenges for poultry health surveillance and disease control. The well-established enteric viruses avian rotavirus A (AvRV-A; *Sedoreoviridae*) and avian orthoreovirus (ARV; *Spinareoviridae*) cause significant economic losses in commercial poultry worldwide (Kunić et al., 2024; Chen et al., 2025). Chicken megrivirus genotype C (ChMeV-C; *Picornaviridae*) has been frequently detected in poultry enteric viromes (Boros et al., 2016), yet its pathogenic role and economic impact remain largely unknown. These viruses share gastrointestinal tropism, fecal–oral transmission, and clinical manifestations such as diarrhea, dehydration, and reduced feed efficiency, particularly in young birds, and they are also associated with impaired nutrient absorption, immune suppression, and stunted growth (Boros et al., 2014; Devaney et al., 2016; Gallego et al., 2022; Nour et al., 2023). Beyond these shared features, AvRV-A is a major cause of enteritis and runting–stunting syndrome (RSS) in broilers, with disease severity influenced by viral strain variations, environmental factors, and coinfections (Gallego et al., 2022). In contrast, ARV causes systemic infections manifesting as viral arthritis, tenosynovitis, and intestinal malabsorption, all of which contribute to skeletal damage and immune dysfunction, thereby predisposing birds to secondary microbial infections (Nour et al., 2023; Rafique et al., 2024). While definitive evidence for a causal role is lacking, ChMeV-C has been implicated in disruption of gut health and growth performance (Boros et al., 2014; Devaney et al., 2016; Nwokorogu et al., 2024). Overall, co-circulation and coinfections involving multiple enteric viruses are common in poultry and can exacerbate disease outcomes while undermining vaccination responses (Pantin-Jackwood et al., 2007; Chen et al., 2022; Grafl et al., 2025).

The segmented double-stranded RNA (dsRNA) genome of AvRV-A consists of 11 segments. These encode six structural proteins—VP1–VP4 (RNA polymerase complex and protease), VP6 (inner capsid), and VP7 (outer capsid)—and up to six nonstructural proteins (NSP1–NSP5/6). Notably, NSP5 and NSP6 are co-encoded on segment 11 and play roles in replication and host interactions (Matthijnssens et al., 2008; Trojnar et al., 2013; Hoxie and Dennehy, 2021). With global distribution, AvRV-A cases have been documented across the Americas, Europe, and Asia, including reports from India, where multiple genotypes and reassortant constellations have been described (Dhama et al., 2015; Gallego et al., 2022). The Rotavirus Classification Working Group (RCWG) nomenclature designates AvRV-A genotypes based on all 11 genome segments, expressed as the constellation Gx-P[x]-Ix-Rx-Cx-Mx-Ax-Nx-Tx-Ex-Hx, corresponding to VP7, VP4, VP6, VP1, VP2, VP3, NSP1, NSP2, NSP3, NSP4, and NSP5/6, respectively (Matthijnssens et al., 2008). The strains are genetically diverse, driven by reassortment between coinfecting strains and rare intra-segment recombination, most often in VP4 and VP7 (Hoxie and Dennehy, 2020; Suzuki et al., 2024). Reports of an AvRV-A strain harboring a VP4 gene closely related to mammalian AvRVs strongly suggest cross-species reassortment (Trojnar et al., 2013; Patzina-Mehling et al., 2020). Because AvRV-A infections are predominantly subclinical, multifactorial, and frequently compounded by enteric coinfections, vaccine development remains challenging, leaving control largely dependent on biosecurity and maternal immunity (Dhama et al., 2015; Kunić et al., 2024).

The non-enveloped ARV has a double-layered capsid enclosing a 10-segmented dsRNA genome (L1–L3, M1–M3, S1–S4), encoding eight structural proteins: λA, λB, λC, μA, μB, σA, σB, and σC, and four nonstructural proteins: μNS, σNS, p10, and p17 (Benavente and Martínez-Costas, 2007; Nour and Mohanty, 2024; Rafique et al., 2024; Liu et al., 2025). Structural proteins include five conserved inner capsid components (λA, λB, λC, μA, and σA) and three variable outer capsid proteins (μB, σB, and σC), the latter influencing host interactions and antigenicity (Benavente and Martínez-Costas, 2007). First isolated in 1954 from chickens with respiratory disease, ARVs were subsequently linked to viral arthritis and are now widely reported in poultry from the Americas and Asia (Fahey and Crawley, 1954; Walker et al., 1972; Rafique et al., 2024; Chen et al., 2025). Sequence variability in the σC gene provides the basis for classifying ARVs into six recognized genotypic clusters (GCI–GCVI) (Liu et al., 2003; Rafique et al., 2024). The S1 segment, which encodes σC, p10, and p17, is also a hotspot for recombination, further contributing to ARV genetic diversity (Hoxie and Dennehy, 2021). σC-driven antigenic divergence may enable emerging field strains to evade immunity conferred by classical vaccines typically derived from GCI reference strains including S1133, 1733, and 2408 (Tang et al., 2016; Palomino-Tapia et al., 2018; Rafique et al., 2024).

The positive-sense ssRNA genome of ChMeV-C (~9.6 kb) encodes a polyprotein cleaved into P1 (structural capsid proteins VP0, VP3, VP1) and P2–P3 (nonstructural proteins 2A–2C and 3A–3D, respectively) involved in replication and assembly (Boros et al., 2014). First identified in poultry in Hungary and the USA, ChMeV-C has subsequently been reported across multiple continents, including Europe (The Netherlands, Switzerland, Hungary), South America (Brazil), Asia (India, Iran), Africa (South Africa), and Oceania (Australia) (Farkas et al., 2012; Boros et al., 2014; Gerber et al., 2019; Kwok et al., 2020; Nwokorogu et al., 2024). Its classification is based on amino acid sequence analysis of the P1 capsid (VP0–VP3–VP1) region and the combined 2C and 3C-D regions (Boros et al., 2014). Among the currently described genotypes, genotype C1 (MeV-C1) is the most frequently reported and geographically widespread, with strains detected in fecal samples from commercial poultry flocks in the aforementioned regions (Boros et al., 2014; Lima et al., 2019; Kwok et al., 2020; Kubacki et al., 2022). Phylogenetic discrepancies between structural and nonstructural regions suggest historical recombination events in ChMeV-C, potentially contributing to genotype diversification and antigenic variation (Boros et al., 2014; Gerber et al., 2019). No licensed vaccines are currently available, leaving control to rely primarily on biosecurity and effective farm management.

This study is part of a broader ntNGS-based initiative to map avian viral pathogens across the IMETA region (India, Middle East, Turkey, and Africa), aimed at generating genomic data to inform vaccination strategies, strengthen biosecurity, and guide targeted interventions. Within this framework, we report the complete genomes of the three genetically distinct enteric viruses described above, from clinical samples of commercial broilers on three farms in Northeast India. To our knowledge, this study provides the first ntNGS-based characterization of concurrent circulation of AvRV-A, ARV, and ChMeV-C in commercial broiler flocks from Kamrup Rural District, Assam, Northeast India, and the findings should be interpreted within this regional context, as India’s marked agroclimatic heterogeneity limits broader extrapolation.

## 2 Materials and methods

### 2.1 Sampling sites and flock metadata

Samples were collected from three commercial broiler farms, anonymized as Farms A, B, and C for confidentiality, located within Kamrup Rural District, Assam, Northeast India. The district spans approximately 3,105 km², with the farms situated roughly 30 km apart in an area dense with poultry operations clustered around major towns and transport routes. At the time of sampling, all flocks exhibited elevated mortality and below-standard body weights. Farm A comprised 19-day-old broilers with a cumulative mortality of 10.6%, whereas Farms B and C had 33-day-old broilers with cumulative mortalities of 25.4% and 14.4%, respectively. The 33-day-old broilers in Farms B and C averaged 1,200 g and 1,100 g, respectively, compared to the regional integration benchmark standard of 1,675 g for that age; average body weight data for flock A were not available. Across the three farms, postmortem examinations revealed pale, enlarged kidneys; hepatomegaly; and distended ureters containing urates, indicative of compromised flock health. Because sampling was based on farm-level composite pools, individual-bird viral loads and within-flock clinical heterogeneity could not be assessed. Vaccination histories of the sampled flocks are provided in **Supplementary Table S1**.

### 2.2 Collection and laboratory processing of clinical samples

To cost-effectively maximize pathogen detection and nucleic acid recovery, cloacal swabs from 50 broilers per farm were pooled in 2 mL of Kylt^®^ Swab Buffer, yielding three farm-level composite pools. For safe transportation and nucleic acid preservation (Kohl et al., 2015; SAN Group, 2021), equal volumes from each pool were combined and spotted onto filter-based AniCards, then dried according to the manufacturer’s instructions (NGS Sampling Kit; SAN Group Biotech GmbH; Emstek, Germany). Total nucleic acids were subsequently extracted from the combined material of the three pools using the Kylt^®^ RNA/DNA Purification Kit. To reduce host-derived (chicken-specific) nucleic acid background and enrich for viral RNA, a proprietary host DNA/RNA depletion protocol from SAN Group was applied, consistent with established viral metagenomics methods (Conceição-Neto et al., 2015; Kohl et al., 2015).

### 2.3 Next-generation sequencing

Extracted RNA was assessed for concentration (Qubit) and for integrity and quality (TapeStation). Complementary DNA (cDNA) was synthesized using random hexamers and reverse transcriptase to capture a broad spectrum of RNA viruses (SAN Group, proprietary procedures). Sequencing libraries were quantified and quality-checked as above, then subjected to paired-end sequencing (2 × 151 bp) on an Illumina MiSeq platform with a 300-cycle Reagent Kit v2, an established configuration for metagenomic ntNGS of microbial communities (Conceição-Neto et al., 2015; Álvarez-Narváez et al., 2025).

### 2.4 Sequence assembly and annotation

Raw sequencing reads were trimmed for adapters and low-quality bases using Trim Galore v0.6.10 (-q 8), and chimeric reads removed with an in-house wrapper tool to prevent artifactual assemblies and segment misassignments. Validation of this step was confirmed by the absence of alternative contig forms for any recovered genome segment. Host-derived (chicken-specific) reads were filtered using BBTools bbduk v39.01 against the *Gallus* reference genome (NCBI RefSeq, current release) to enhance viral detection specificity. The remaining high-quality reads were assembled *de novo* with MEGAHIT v1.2.9 (Li et al., 2015; Paez-Espino et al., 2017), and contigs with significant similarity to known viral genomes in reference databases were retained for further analysis. Quasispecies and minor variants were identified using LoFreq v2.1.5 and allele frequency analysis with bcftools v1.19 (Paez-Espino et al., 2017). Open reading frames (ORFs) and functional genomic elements were annotated in Geneious Prime^®^ v2025.2.1, as previously described (Kariithi et al., 2022).

### 2.5 Phylogenetic analyses

Sequences generated in this study, together with representative sequences retrieved from GenBank via BLASTn or BLASTp searches, were aligned with MAFFT v7.511 (Katoh and Standley, 2013) in the Geneious Prime^®^ platform. Alignments were trimmed in trimAl v1.3 (Capella-Gutiérrez et al., 2009) in *gappyout* mode to reduce the influence of poorly aligned regions. Phylogenetic trees were inferred using the maximum likelihood (ML) method in MEGA X (Kumar et al., 2018), with best-fit substitution models selected automatically based on the corrected Akaike Information Criterion (see tree legends). Each dataset was evaluated with 1,000 bootstrap replicates to assess the robustness of inferred relationships.

### 2.6 Recombination analyses

Putative recombination events in the assembled consensus genome sequences of the Indian AvRV-A, ARV and ChMeV strains were evaluated in RDP4 v4.101 (Martin et al., 2015). Analyses were performed on datasets comprising 86 AvRV-A, 87 ARV, and 76 MeV genotypes A, B, C, and E sequences, incorporating both global and available regional references to maximize detection sensitivity. Eight heuristic recombination detection algorithms (RDP, GENECONV, BootScan, MaxChi, Chimaera, SiScan, LARD, and 3Seq) were employed with a significance threshold of *P ≤* 0.05, Bonferroni multiple correction, and SEQ-GEN parametric simulations. A recombination event was considered confirmed only when identified by at least five algorithms and supported by corrected p-values ≤ 1 × 10^-6^ at inferred breakpoints.

## 3 Results

### 3.1 Composition and taxonomic classification of NGS reads

The ntNGS produced 137,968 quality-filtered read pairs from an initial 412,075 raw reads (≈206,038 read pairs) after trimming and quality control. Of these, 2,049 read pairs (1.49%) mapped to host (chicken) genome. Viral reads were assigned as follows: AvRV-A (8,845 read pairs; 6.41%), ARV (7,408 read pairs; 5.37%), and ChMeV-C (924 read pairs; 0.67%), with complete genomes recovered as detailed in subsections 3.2–3.4. A smaller proportion (127 read pairs; 0.09%) mapped to infectious bursal disease virus (IBDV; Gumboro), assembling into eight short contigs (151–473 nt) across segments A and B; however, genome-level analysis was not pursued due to insufficient coverage. Bacterial 16S and 23S rRNA fragments were also detected from *Gallibacterium anatis* (n = 323) and *Campylobacter jejuni* (n = 158), but these are not further discussed in this study. The remaining reads consisted mainly of unclassified environmental sequences and low-abundance bacterial fragments, which were beyond the scope of this study.

### 3.2 Chicken megrivirus genotype C (ChMeV-C; *Picornaviridae* family)

**Figure 1:**
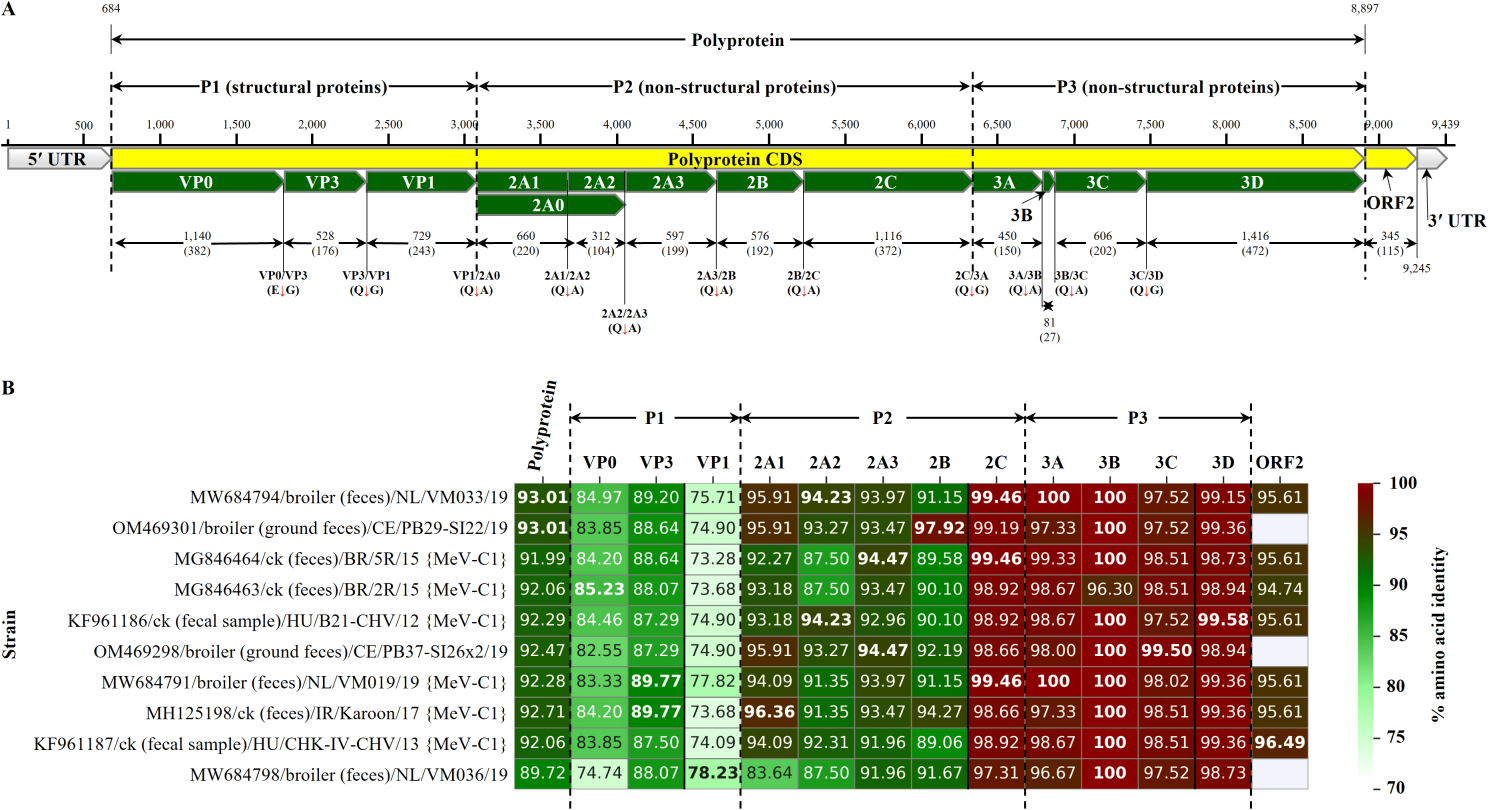
Genome organization and comparative amino acid (aa) identities of chicken megrivirus strain ChMeV-C/broiler/IN/A2323728-003/23 identified in this study. **(A)** Schematic representation of the complete genome structure of the Indian strain. The polyprotein coding region (CDS) is shown along with the 5′ and 3′ untranslated regions (UTRs), the putative ORF2, and individual predicted proteins. The length of each genomic region is indicated in nucleotides (nt), and the predicted mature viral proteins resulting from post-translational polyprotein processing are annotated with aa lengths in parentheses; cleavage sites are marked with red arrows, with motifs (e.g., Q↓G, Q↓A, E↓G) indicated. **(B)** Heatmap comparing aa sequence identity between the Indian strain and the top 10 BLASTp-based hits of representative megriviruses from GenBank. Identity values are shown for the complete polyprotein, individual structural and nonstructural proteins, and ORF2. Protein regions are grouped by functional categories P1 (structural), P2 and P3 (nonstructural), and ORF2, and are visually separated by vertical black dotted lines. Color gradients range from red (highest identity) to white (lowest), with green representing intermediate values; gradients are scaled independently for each region to enhance contrast. Gray cells indicate missing data (i.e., regions absent in that strain), and bolded values highlight the highest identity observed within each region. Strain labels indicate GenBank accession number, host species, country of origin (two-letter ISO code), strain designation, and year of sample collection.

A complete ChMeV-C genome was *de novo* assembled as a single 9,439-nt gap-free contig (excluding the poly(A) tail), with a median coverage depth of 46× and a GC content of 44.1%. Designated ChMeV-C/broiler/IN/A2323728-003/23, the genome is consistent with members of the *Megrivirus* genus and comprises a 683-nt 5′ untranslated region (UTR), an 8,214-nt polyprotein-encoding ORF (2,737 amino acids [aa]), a 345-nt putative ORF2, and a 195-nt 3′ UTR (**Figure 1A**). Pairwise aa comparisons showed high overall similarity to reference ChMeV-C strains from multiple geographic regions (**Figure 1B**), with the highest identities to strains from the Netherlands (Kwok et al., 2022), Switzerland (Kubacki et al., 2022), Hungary (Boros et al., 2014), Brazil (Lima et al., 2019), and Iran (strain Karoon; GenBank: MH125198). The P3 region (3A–3D) was most conserved (97.3–100% aa identity), followed by protein 2C (~99%) and ORF2 (94.7–96.5%). In contrast, P2 (2A1–2B) and P1 proteins were more variable, with identities of 83.6–96.4% and 84.2–89.8%, respectively. The overall polyprotein identity ranged from 89.7% to 93.0%, with all top matches derived from fecal samples, including six from the widely distributed GC1.

Five nonsynonymous single nucleotide polymorphisms (SNPs) were detected in minor viral populations (**Table 1**; **Supplementary Table S2**), including one each in VP0 (V294G), VP1 (R68P), and ORF2 (T23I), and two in the RNA-dependent RNA polymerase 3D (RdRp; T140S and H405N). Variant allele frequencies ranged from 11.0% to 24.0%, a level of intra-strain diversity typical of RNA viruses (Singh et al., 2023). Although ChMeV-specific SNP data remain limited, substitutions in RdRp and structural proteins such as VP1 and VP0 have been linked to virulence or immune escape in other picornaviruses, and variability in VP1 and 3D suggests recurrent mutation hotspots (Boros et al., 2014; Gerber et al., 2019).

**Table 1:** Nonsynonymous SNPs found in the avian rotavirus A strain AvRV-A/broiler/IN/A2323728-003/23/G19P[31], avian orthoreovirus strain Reo/broiler/IN/A2323728-003/23, and chicken megrivirus strain ChMeV-C/broiler/IN/A2323728-003/23 identified in the current study.

Virus strain	Genomic region	Description	nt position	Consensus seq.		Variant		Read coverage depth at variant position	Variant frequency	Coding effect
				Codon	aa residue	Codon	aa residue			
ChMeV-C/broiler/IN/A2323728-003/23	VP0	VP0 (capsid precursor)	1,564	G**T**A	Val	G**G**A	Gly	63×	11.10%	V294G
	VP1	VP1 (major capsid protein)	2,542	C**G**T	Arg	C**C**T	Pro	39×	15.40%	R68P
	3D	3D (RNA-dependent RNA polymerase)	7,896	**A**CT	Thr	**T**CT	Ser	60×	16.70%	T140S
			8,691	**C**AT	His	**A**AT	Asn	91×	24.20%	H405N
	putative ORF2	protein of unknown function	8,967	A**C**C	Thr	A**T**C	Ile	63×	12.70%	T23I
Reo/broiler/IN/A2323728-003/23	L1	λA (core shell scaffold)	753	**G**AT	Asp	**C**AT	His	160×	10.0%	D245H
			3,531	**A**CG	Thr	**T**CG	Ser	146×	11.0%	T1171S
	L3	λC (mRNA capping enzyme)	1,946	C**T**G	Leu	C**C**G	Pro	77×	11.7%	L645P
	M1	μA (core NTPase)	2,152	G**G**T	Gly	GAT	Asp	19×	26.3%	G713D
	M3	μNS (viroplasm matrix protein)	648	AT**G**	Met	AT**A**	Ile	213×	17.8%	M208I
			946	**T**TT	Phe	**C**TT	Leu	144×	20.8%	F308L
	S3	σA (RNA-binding core protein)	562	**T**CT	Ser	**G**CT	Ala	158×	12.0%	S178A
			616	**C**CT	Pro	**T**CT	Ser	193×	11.4%	P196S
	S4	σNS (RNA-binding cofactor)	994-995	A**AA**	Lys	A**GG**	Arg	45×	17.8%	K324R
AvRV-A/broiler/IN/A2323728-003/23/G19P[31]	2	VP2 (inner capsid scaffold)	2,571	**G**CC	Ala	**A**CC	Thr	70×	20.0%	A852T
	8	NSP2 (viroplasm RNA binding protein)	545	**T**GG	Trp	**C**GG	Arg	130×	11.5%	W168R
	9	VP7 (outer surface glycoprotein)	155	G**C**T	Ala	G**T**T	Val	181×	31.5%	A37V

Variants were filtered using the following thresholds: minimum frequency of 10%, minimum read depth of 10×, and maximum strand bias of 40. The variations in the nucleotide codons are underlined. The complete list of synonymous and nonsynonymous polymorphisms is provided in **Supplementary Table S2**.

**Figure 2:**
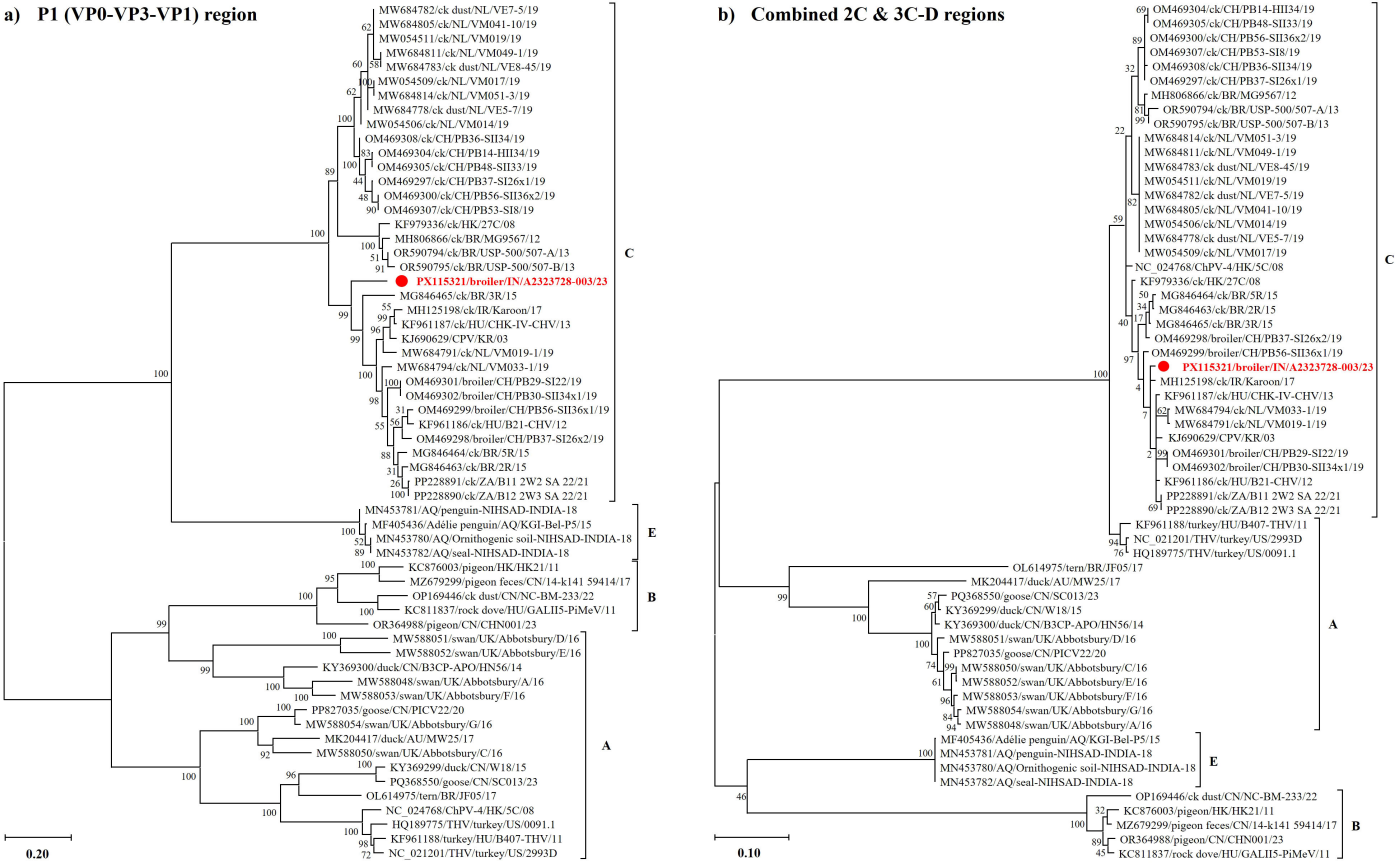
Phylogenetic clustering of the Indian strain ChMeV-C/broiler/IN/A2323728-003/23 identified in this study (highlighted in bold red) based on structural and nonstructural protein regions. Phylogenetic trees were inferred from translated amino acid sequences of **(A)** the P1 region (VP0–VP3–VP1) and **(B)** the combined 2C and 3CD regions of representative megriviruses. The Indian strain clusters within the MeV-C clade in both trees. In the P1 tree, it forms a distinct and well-supported sublineage (bootstrap = 99%) separate from other MeV-C strains from Europe (Hungary, Switzerland, and the Netherlands), South America (Brazil), and South Africa. This pattern is not recapitulated in the 2C–3CD tree, where clustering is less resolved and bootstrap-supported. The ML trees were constructed using the LG+G+I+F model for the P1 region and the LG+G model for the 2C–3CD region, based on 60 sequences aligned over 761 and 1,030 aa positions, respectively. Sequence labels include GenBank accession numbers, host species, country of origin (two-letter ISO code), strain name, and year of sample collection.

Phylogenetic trees based on aa sequences of the P1 (VP0–VP3–VP1) and combined 2C and 3C-D regions (**Figures 2A, B**, respectively) placed the Indian ChMeV-C strain within the MeV-C clade. In the P1 tree, it formed a distinct, well-supported sublineage (bootstrap = 99%) closely related to Swiss, Dutch, Brazilian, and Hungarian strains. In contrast, the 2C–3CD tree clustered it with these strains but with low bootstrap support (2%), reflecting the high conservation of this region, particularly across the P3 proteins (3A–3D; 96.3–100%) and 2C (97.3–99%). The strain also grouped with two South African C2 viruses (B11_2W2_SA_22 and B12_2W3_SA_22; GenBank: PP228891 and PP228890) from asymptomatic broilers in KwaZulu-Natal (Nwokorogu et al., 2024), suggesting shared ancestry and possible intercontinental dissemination. Although detected in birds experiencing health and performance challenges, these findings do not establish a causal role for ChMeV-C in disease based on the present study.

### 3.3 Avian orthoreovirus (ARV; *Spinareoviridae* family)

The 7,408 ARV-specific reads were *de novo* assembled into 10 gap-free contigs representing the complete genome segments, with median coverage depths of 89× to 163× (**Table 2**). Designated here as Reo/broiler/IN/A2323728-003/23, the genome architecture of the Indian strain is consistent with other ARVs (Benavente and Martínez-Costas, 2007), with all segments containing the conserved terminal sequences of the positive-sense RNA strand: GCUUUU[U/C] at the 5′ end and UCAUC at the 3′ end. Segment lengths ranged from 1,196 to 3,958 bp, aligning with published coding sequence (CDS) sizes (Benavente and Martínez-Costas, 2007; Matthijnssens et al., 2022).

**Table 2:** Genome segment organization and assembly metrics of the Indian avian orthoreovirus strain Reo/broiler/IN/A2323728-003/23 identified in the current study.

Segment	Encoded protein	Published CDS length range (bp) ^a^	*de novo* assembly		CDS			Closest BLASTn hit			GenBank accession number
			Consensus seq. length (bp) ^b^	Median coverage depth	Coordinates	nt	aa	Strain description	Genotypic group (GC) ^c^	Protein (aa) identity	
L1	λA (core shell scaffold protein)	3,958–3,961	3,958	131×	21–3,902	3,882	1,293	KX398282/chicken/Hungary/3457-M-11/11	III	99.23%	PX115322
L2	λB (RNA-dependent RNA polymerase)	3,829–3,860	3,829	136×	14–3,793	3,780	1,259	OQ731572/turkey/Hungary/209/16	III	97.93%	PX115323
L3	λC (mRNA capping enzyme)	3,870–3,910	3,907	109×	13–3,870	3,858	1,285	KC865788/chicken/Hungary/T1781/12	III	96.50%	PX115324
M1	μA (core NTPase)	2,263–2,270	2,264	89×	15–2,213	2,199	732	KX398285/chicken/Hungary/3457-M-11/11	III	97.81%	PX115325
M2	μB (outer capsid precursor)	2,150–2,180	2,158	110×	30–2,060	2,031	676	PQ490771/chicken/China/FJ/ZYQ/2402/24	unclassified	98.22%	PX115326
	μBN (N-terminal cleavage product)				30–155	126	42			100%	
	μBC (C-terminal cleavage product)				156–2,060	1,905	634			98.11%	
M3	μNS (Viroplasm matrix protein)	1,980–2,000	1,996	163×	25–1,932	1,908	635	KX398287/chicken/Hungary/3457-M-11/11	III	95.91%	PX115327
S1	p10 (FAST protein)	1,600–1,650	1,645	129×	23–322	300	99	PQ381511/broiler/USA/GA/22-835/23	IV	89.58%	PX115328
	p17 (membrane regulator)				294–734	441	146	OR612129/chicken/USA/Alabama/21	IV	84.25%	
	σC (spike/receptor-binding protein)				631–1,611	981	326	LC605824/chicken/Japan/CS-108	IV	88.04%	
S2	σA (RNA-binding core protein)	1,300–1,350	1,324	102×	16–1,266	1,251	416	KX398319/chicken/Hungary/17203-M-06/06	IV	99.52%	PX115329
S3	σB (outer capsid antigenic protein)	1,200–1,250	1,202	158×	31–1,134	1,104	367	MF686702/chicken/South Korea/K738/14	II	97.00%	PX115330
S4	σNS (RNA-binding cofactor)	1,150–1,200	1,173	130×	24–1,127	1,104	367	KX398241/chicken/Hungary/284-V-06/06	III	97.82%	PX115331

**Figure 3:**
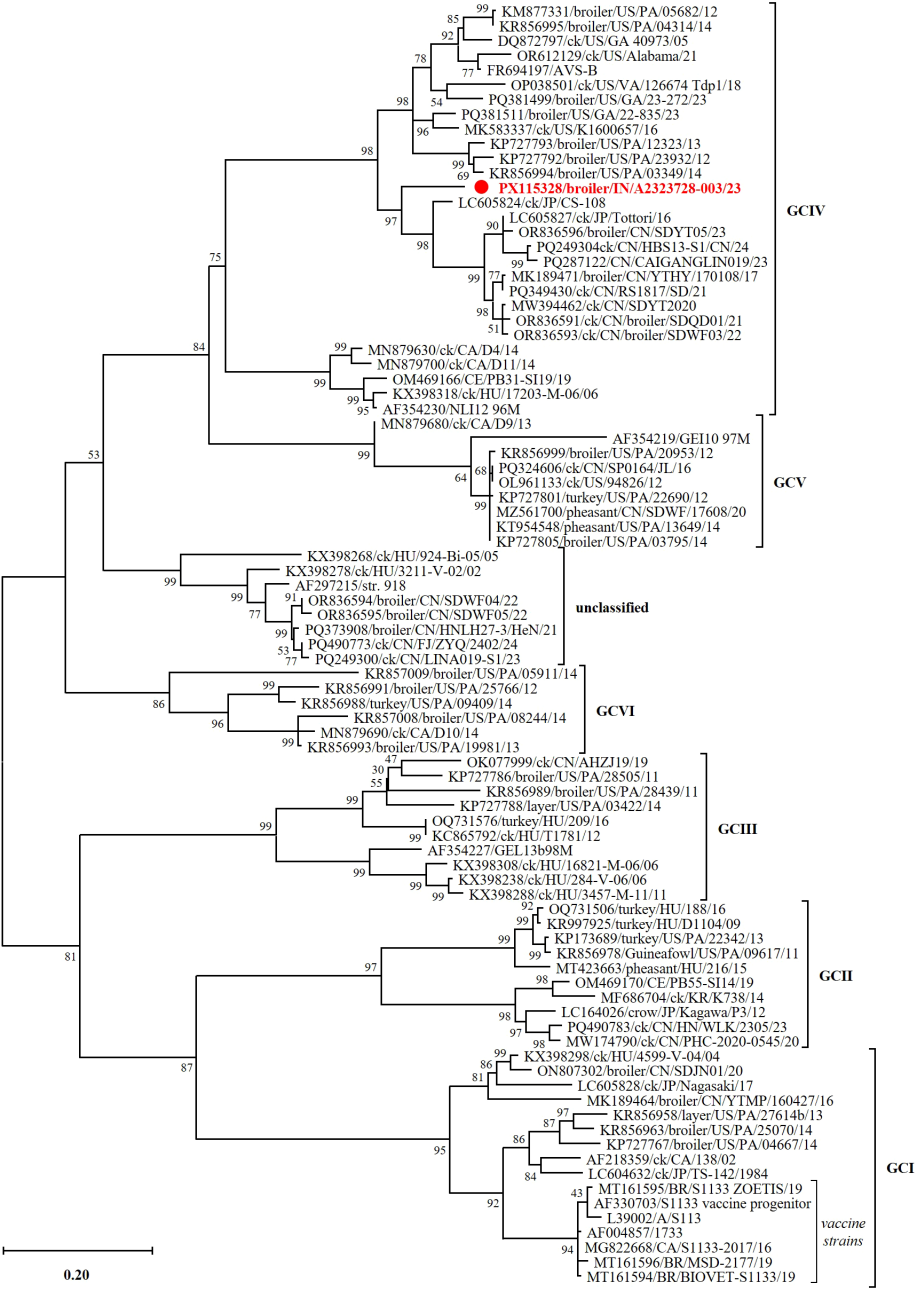
Phylogenetic placement of the Indian ARV strain Reo/broiler/IN/A2323728-003/23 identified in this study (highlighted in bold red), grouping with Chinese and Japanese strains within GCIV but forming a separate and well-supported lineage (97% bootstrap support). Analysis was based on the amino acid sequences of the sigma C (σC) protein and conducted using the JTT+G+I+F model. The final dataset comprised 87 sequences aligned over 300 aa positions. Sequence labels indicate GenBank accession numbers, host species, country of origin (two-letter ISO code), strain name, and year of sample collection.

The assembled genome comprises complete CDSs for all 10 segments, with high median coverage depths (89×–163×). BLASTp analysis showed that most segments shared high amino acid identity (96.5–99.5%) with GCIII strains from Hungary, whereas the S1-encoded p10, p17, and σC clustered with GCIV strains despite lower identities of 89.6%, 84.3%, and 88.0%, respectively. This genotypic assignment is supported by the phylogenetic analysis shown in **Figure 3**.

^a^Published segment coding sequence (CDS) length ranges are based on established values (Benavente and Martínez-Costas, 2007; Matthijnssens et al., 2022).

^b^Consensus sequence length and median coverage depth are based on *de novo* assembly of NGS reads as described in the text.

^c^Genotypic grouping was assigned based on the S1 sigma C (σC) sequence clustering within established ARV GCs (see phylogenetic trees in **Figure 3**; **Supplementary Figure S1g**).

Polymorphisms from minor viral populations included nine nonsynonymous SNPs (**Table 1**; **Supplementary Table S2**) that resulted in aa substitutions across multiple segments: L1 (λA, core shell protein: D245H), L2 (λB, RdRp: T1171S), L3 (λC, core turret protein: L645P), M1 (μA, core NTPase: G713D), M3 (μNS, viroplasm matrix protein: M208I, F308L), S3 (σB, outer capsid antigen: S178A, P196S), and S4 (σNS, RNA-binding protein: K324R). Variant allele frequencies ranged from 10.0% to 26.3%. Similar variations have been associated with strain-specific virulence and immune modulation in ARV (Ayalew et al., 2022; Chen et al., 2025).

BLASTp analysis of aa sequences showed that segment S1, encompassing complete p10, p17, and σC proteins, most closely matched ARVs from the USA and Japan but with comparatively low identities—89.58% (p10), 84.25% (p17), and 88.04% (σC) (**Table 2**). Despite this, σC placed the strain within GCIV by established classification criteria. Six other segments (L1, L2, L3, M1, M3, and S4) were most similar (95–99% identity) to Hungarian strains (17203-M-06, 3457-M-11, T1781, and 209). Segment S3 showed the highest similarity (97%) to a South Korean strain (K738), while M2 (encoding μB, μBN, and μBC) shared 98.22%, 100%, and 98.11% identity, respectively, with a Chinese strain (FJ/ZYQ/202402) that remains unclassified. These segment-wise differences indicate a genomic mosaic pattern consistent with historical reassortment among geographically diverse ARVs.

Phylogenetic analysis of σC aa sequences placed the Indian strain within GCIV, forming a distinct subcluster with Chinese and Japanese strains, separate from US-derived lineages (**Figure 3**). This subcluster was strongly supported (97% bootstrap), indicating marked divergence from other South and East Asian counterparts. Segment-wise trees (**Supplementary Figures S1a–j**) revealed heterogeneous clustering. In the S1 tree (**Supplementary Figure S1g**), the strain grouped with Chinese (RS1817/SD/21, SDYT2020) and US (PA/05682/12, Alabama/21) GCIV viruses, supported by high bootstrap values, whereas Canadian (CA/D4/14, CA/D11/14) and European (HU/T1781/12, CE/PB31-SI19/19) GCIV strains formed a separate subclade. Strong bootstrap support for distinct placement was observed in L1 (99%) and L3 (100%) trees, moderate in M1 (66%), while the strain clustered with the South Korean K738 in M3 and S3. L2 (λB) and S2 (σA) showed broader clustering with GCI–GCIV and unclassified strains without clear separation. Overall, despite segmental divergence, the Indian strain is best classified as GCIV based on σC and S1 phylogenies, with its distinct clustering in other segments suggesting ongoing genomic diversification among circulating ARVs.

### 3.4 Avian rotavirus A (AvRV-A; *Sedoreoviridae* family)

*De novo* assembly of 8,845 AvRV-A–specific reads yielded 11 gap-free contigs representing complete genome segments, with high median coverage (149×–298×) and segment lengths consistent with published AvRV-A ranges (Hoxie and Dennehy, 2021). The Indian strain encodes six structural proteins (VP1–VP4, VP6, VP7) and six nonstructural proteins (NSP1–NSP6), reflecting the conserved genome organization of group A rotaviruses. The assembled segments are summarized in **Table 3**, including nt lengths, CDS coordinates, and genotype assignments based on RCWG criteria (Matthijnssens et al., 2011). By these criteria, nt identities exceeded genotype thresholds for 10 of 11 segments, yielding the constellation G19–P[31]–I11–R6–C6–M7–A16–N6–Tx–E10–H8 (NSP3 provisionally Tx), and the strain is designated AvRV-A/broiler/IN/A2323728-003/23/G19P[31]. Segment 7 (NSP3) showed 82.77% nt identity, slightly below the 85% cut-off for T8 assignment, and was therefore provisionally designated Tx, reflecting insufficient evidence for assignment to an established T genotype or formal designation of a novel genotype. Closest BLASTn matches were mainly AvRV-A strains from Germany (five segments), followed by Switzerland and Ireland (two each), and Japan and India (one each).

**Table 3:** Sequence analysis of the Indian avian rotavirus A strain AvRV-A/broiler/IN/A2323728-003/23/G19P[31] in the current study.

Segment	Encoded protein	Consensus seq. length (bp) ^a^	CDS		Median coverage depth	Closest BLASTn Hit to complete CDS			Genotype assignment		GenBank accession number
			Coordinates (nt range)	Length (aa residues)		Strain description	Genotype	% nt identity	% nt identity cut-off	Assigned genotype	
1	VP1 (RNA-dependent RNA polymerase)	3,305	19–3,288	1,089	159×	LC088125/Ireland/chicken-tc/Ch-1/79/G19P[30]	R6	90.61%	83%	R6	PX115332
2	VP2 (inner capsid)	2,732	18–2,705	895	156×	OM469186/CH/PB41-SI14/19	C6	92.52%	84%	C6	PX115333
3	VP3 (RNA capping enzyme)	2,583	50–2,539	829	149×	LC785327/chicken/Japan/Kagoshima-877-3/20/G19P[30]	M7	89.97%	81%	M7	PX115334
4	VP4 (outer capsid spike)	2,351	10–2,322	770	177×	MN365937/chicken/Germany/06V0661G1/06/P[31]	P[31]	96.76%	80%	P[31]	PX115335
	VP8*		10–696	229				97.79%			
	VP5*		739–2,322	527				96.72%			
5	NSp1 (interferon antagonist)	2,117	39–1,772	577	164×	MN365916/chicken/Germany/04V0027G6/05	A16	91.29%	79%	A16	PX115336
6	VP6 (intermediate capsid)	1,348	24–1,217	397	253×	DQ096805/Germany/02V0002G3	I11	94.64%	85%	I11	PX115337
7	NSp3 (translation shutoff protein)	1,088	77–991	304	194×	MN365940/chicken/Germany/06V0661G1/06	T8	82.77%	85%	Tx*	PX115338
8	NSp2 (viroplasm RNA-binding)	1,042	46–993	315	288×	MN365908/chicken/Germany/02V0002G3/02	N6	95.89%	85%	N6	PX115339
9	VP7 (outer capsid glycoprotein)	1,066	50–1,039	329	168×	PP348823/chicken/India/249/MIB/CVAS/PKD/23	G19	98.78%	80%	G19	PX115340
10	NSp4 (ER translocator)	724	41–547	168	232×	OM469201/Switzerland/PB32-SII33/19	E10	93.49%	85%	E10	PX115341
11	NSp5/6 (replication complex scaffolding phosphoprotein)	699	22–648	208	298×	LC088137/Ireland/chicken-tc/Ch-1/79/G19P[30]	H8	93.78%	91%	H8	PX115342
	NSp5* (viroplasm component)		22–618	199							
	NSp6* (transmembrane viroporin)		404–613	70							

The complete genome comprises all 11 CDSs with high median coverage depths (149×–298×). Genotype assignments were based on BLASTn analysis and nucleotide identity thresholds established by the Rotavirus Classification Working Group (Matthijnssens et al., 2011), classifying the strain as G19–P[31]–I11–R6–C6–M7–A16–N6–Tx–E10–H8. While most segments showed high nucleotide identity to European or Asian reference strains, segment 7 (NSP3) showed only 82.8% identity, below the 85% RCWG threshold, resulting in its provisional designation as genotype Tx. These classifications are supported by phylogenetic analyses presented in **Supplementary Figure S2**.

VP8* and VP5* are trypsin-mediated proteolytic cleavage products (N-terminal (receptor-binding) and C-terminal (membrane-penetration), respectively) of the spike protein VP4.

NSP5* and NSP6* are generated from segment 11 by direct translation from the primary open reading frame (ORF) and an alternative overlapping ORF, respectively.

^a^Consensus sequence length and median coverage depth are based on *de novo* assembly of NGS reads as described in the text.

Phylogenetic analysis of all 11 genome segments (**Supplementary Figures S2a–k**) corroborated the genotype assignments of the Indian strain. In the VP7 and VP4 trees, it clustered closely with recently reported Indian G19 strains (e.g., MIB/CVAS/PKD/23; GenBank: PP348822–PP348825) and with German P[31] strains from 2006, confirming its G19P[31] classification under the binary system. Several internal segments, including VP6 (I11), VP1 (R6), VP2 (C6), and NSP3 (Tx), showed distinct phylogenetic placements with moderate to high bootstrap support (69%–100%). VP3 grouped with M7 strains from Japan, South Korea, and Ireland, whereas NSP1 was most closely related to A16 strains from Germany, Japan, and Switzerland but remained genetically distinct (97% bootstrap). NSP4 and NSP5/6 exhibited moderate divergence within E10 and H8, supported by 74% and 57% bootstrap values, respectively. Collectively, these segment-specific placements underscore the genetic distinctiveness of the Indian strain.

Eight SNPs from minor viral populations were identified (**Table 1**; **Supplementary Table S2**), including three nonsynonymous substitutions in VP2 (core shell protein: A852T), VP7 (outer capsid glycoprotein: W168R), and NSP2 (RNA-binding protein: A37V). The remaining five were synonymous, located in VP1 (RdRp), VP2, VP3 (RNA capping enzyme), VP6, and VP7. Variant allele frequencies ranged from 11.1% to 40.1%. This diversity, spanning both structural and replication-associated proteins, has been linked to host-specific adaptations in AvRV-A and may influence cross-species infectivity (Falkenhagen et al., 2022).

### 3.5 Recombination analyses

No credible intra-segment recombination events were detected in any genome segment of the Indian AvRV-A, ARV, or ChMeV strains using RDP4. All potential signals were excluded based on one or more of the following criteria: (i) corrected p-values above the 1 × 10^-6^ threshold and/or support from fewer than five RDP4 detection algorithms; (ii) inability to confidently identify one or both breakpoints; or (iii) >30% probability that one or both parental strains were themselves recombinants.

## 4 Discussion

This study confirms the co-circulation of three distinct enteric viruses (AvRV-A, ARV, and ChMeV-C) in commercial broiler flocks in Northeast India. Although the detected IBDV is primarily lymphotropic rather than enteric, its concurrent presence suggests exposure of birds to both immunosuppressive and enteric pathogens, a combination likely to exacerbate susceptibility and modulate viral shedding (Schat and Skinner, 2014); however, the potential contribution of IBDV was not further evaluated within the scope of the present study given the available sequence data. Flock-level records showed elevated mortality and reduced growth performance, suggesting ongoing but largely unrecognized viral transmission in the study flocks. These findings highlight the frequent occurrence of enteric viral coinfections, even in vaccinated flocks, underscoring the need to interpret viral circulation in the broader context of farm-level immunological resilience and biosecurity. Although the flocks were vaccinated against Newcastle disease and Gumboro, these vaccines do not directly protect against enteric viruses; however, their effectiveness can be compromised by maternally derived antibodies, antigenic mismatch, or suboptimal delivery, leaving birds immunologically vulnerable and more susceptible to enteric viral infections (Berg, 2000; Müller et al., 2003).

All three farms were contracted under a single integrator, implying shared chick and feed supply chains, although no records indicated direct exchanges of birds or feed among them. The pooled samples confirmed that birds were exposed to or actively shedding these pathogens. While cost-effective for providing a flock-level snapshot and detecting mixed infections despite individual variation, pooled sampling lacks individual-level resolution and cannot determine the specific contribution of each virus to flock health (Van Borm et al., 2021; Kwok et al., 2022). All genome segments were recovered as single, gap-free contigs with consistent coverage and no alternate forms, supporting the consensus sequences as the predominant circulating variants. Minor allele variants were observed across all three detected enteric viruses, but short read lengths prevented phasing of low-frequency variants and obscured whether they reflected true co-circulating strains or quasispecies fluctuations, a limitation that future individual-level studies could address. Consistent with this scope, metabolic, nutritional, and toxicological assessments were not performed, as the study focused exclusively on ntNGS-based viral detection and genomic characterization.

This study presents one of the first complete ChMeV-C1 genome reports from India, contributing to evidence of its widespread detection in global poultry flocks (Gerber et al., 2019; Kubacki et al., 2022; Kwok et al., 2022). While its pathogenic role remains unclear, its co-detection with ARV and AvRV-A in this study illustrates the complexity of viral communities that can affect gut health and immune function. In a metagenomic comparison of healthy and RSS-affected flocks, Kubacki et al. (2022) reported ARV and AvRV-A as predominant viruses, with MeVs more frequently detected in RSS-affected birds, suggesting a role in disease severity. Similarly, Kang et al. (2012) documented coinfection with chicken astrovirus and AvRV-A that was associated with intestinal lesions and elevated mortality, highlighting the compounded effects of enteric viral synergy on poultry health. The identification of five nonsynonymous minor allele SNPs in VP0, VP1, ORF2, and the RdRp (3D) of the Indian ChMeV-C strain suggests ongoing evolutionary dynamics potentially shaped by selection pressures. Notably, aa substitutions such as R68P in VP1 and H405N in 3D, also found in Hungarian and Brazilian strains (Boros et al., 2014; Gerber et al., 2019), may represent recurrent evolutionary hotspots. The phylogenetic placement of the Indian ChMeV-C strain within a distinct sublineage alongside European, South American, and African strains suggests regional diversification within the globally distributed genotype C1, particularly in structural genes where clustering was strongly supported. These findings align with global reports of widespread detection of ChMeV-Cs in broiler systems, including in both healthy and malabsorption-affected flocks (Lima et al., 2019; Kubacki et al., 2022; Kwok et al., 2022; Nwokorogu et al., 2024), and underscore the potential for long-distance dissemination of genetically related strains. The unique phylogenetic placement of the Indian strain highlights ongoing regional diversification within the C1 lineage and emphasizes the need for sustained metagenomic surveillance to monitor emerging variants and their impacts on flock health and productivity.

The genomic characterization of the Indian ARV strain highlights the complexity of ARV evolution in intensive commercial poultry systems, where high bird density, rapid turnover, and regional trade likely facilitate viral diversification and reassortment (Rafique et al., 2024; Chen et al., 2025). Although reassortment among dsRNA viruses is constrained by packaging compatibility (McDonald et al., 2016), the phylogenetic incongruence observed across multiple genome segments suggests that the Indian strain constitutes a genomic mosaic. Such mosaicism may influence antigenicity and virulence, complicating vaccine design. Several segments showed the highest similarity to ARV strains from Hungary, China, South Korea, Japan, and the USA, consistent with reports implicating international trade and biological materials in the global dissemination of ARVs (Tang et al., 2016; Palomino-Tapia et al., 2018). Within the IMETA (India, the Middle East, Turkey, and Africa) region, movement of poultry products may further facilitate the emergence of genetically diverse ARV strains. While classified as GCIV based on S1 and σC sequences, the broader divergence of the Indian strain raises concerns about potential vaccine mismatch. The nine nonsynonymous minor allele SNPs detected across key structural and replication-associated proteins (λA, λB, λC, μA, μNS, σB, and σNS) may reflect emerging functional differences among field isolates (Ayalew et al., 2022; Chen et al., 2025). However, the use of conservative variant-calling thresholds may have limited the detection of low-frequency variants or minor strain populations below this threshold. These findings warrant functional validation to clarify their biological and clinical significance. Most licensed ARV vaccines, including GCI strains S1133, 1733, 2408, and 2177 (Rafique et al., 2024), and their limited antigenic overlap with divergent strains such as the Indian isolate, underscore the need for regionally informed vaccine updates and strengthened genomic surveillance. Direct sequence comparisons with locally used vaccine seed strains were not possible because reference sequences were unavailable in public databases at the time of analyses. While global ARV diversity is shaped by frequent reassortment, no credible recombination signals were detected in the Indian ARV strain, possibly reflecting the limited availability of comprehensive regional reference genomes needed to detect such events.

The Indian AvRV-A strain characterized in this study exhibits a genotype (G19–P[31]–I11–R6–C6–M7–A16–N6–Tx–E10–H8) closely resembling configurations reported in poultry strains from Germany and Brazil (Trojnar et al., 2013; Beserra et al., 2014), suggesting a conserved genomic backbone among geographically dispersed avian AvRV-A strains. However, the phylogenetic distinctiveness of several segments, including VP6, VP1, VP2, and NSP3, possibly reflects regional diversification within otherwise conserved genotypes. Similar to ARV, no credible recombination signals were detected in the Indian AvRV-A strain, despite global reports of reassortment, likely reflecting the limited availability of regional reference genomes needed to detect such events. These findings address a critical knowledge gap in India, where genomic data on poultry AvRV-A remain scarce. Earlier studies have primarily focused on RVD or partial genotyping; to our knowledge, this is among the few full-genome-level analyses of Indian AvRV-As (Savita et al., 2008). The observed divergence, particularly in NSP3 (provisionally designated Tx), alongside variability in clustering patterns of structural and nonstructural proteins, may reflect ongoing viral evolution and diversification, consistent with findings in other AvRV-A genomic studies (López et al., 2016). This unassigned status of NSP3 highlights gaps in current genotype frameworks and the need for expanded sequence databases. Furthermore, eight SNPs, including three nonsynonymous SNPs in VP2, VP7, and NSP2, suggest potential host adaptation or relaxed purifying selection. This aligns with evidence of positive selection in key structural and nonstructural proteins, such as VP7 and NSP4, observed in avian and mammalian rotavirus strains (Song and Hao, 2009; Hoxie and Dennehy, 2020; Nichols et al., 2024; Suzuki et al., 2024). Taken together, the genetic distinctiveness and indications of evolutionary change in the Indian AvRV-A strain highlight the need for sustained regional genomic surveillance and vaccine-informed interventions to safeguard poultry health.

## 5 Conclusions

This study is the first to document the co-circulation and complete genomic profiling of AvRV-A, ARV, and ChMeV-C in Indian broiler flocks experiencing health and performance challenges. The detection of genetically diverse enteric viruses, even in vaccinated flocks, underscores the complexity of viral circulation in production systems. While causality cannot be assigned, the genomic data provide actionable baselines for surveillance and vaccine strain selection. Observed variations, including nonsynonymous polymorphisms and distinct phylogenetic clustering patterns, indicate ongoing microevolution with potential implications for virulence, host adaptation, and vaccine efficacy. Metagenomic ntNGS remains a vital tool for revealing pathogen diversity, characterizing emerging variants, and informing region-specific control strategies. Sustained genomic surveillance in high-density poultry production areas is essential for early detection of variants and for reducing vaccine failure risks. Such measures are critical to preventing localized outbreaks from escalating into regional or transboundary threats to poultry health and food security.

## Data Availability

The complete genome sequences generated in this study have been deposited in GenBank under the following accession numbers: • Strain ChMeV-C1/broiler/IN/A2323728-003/23: complete genome (PX115321). • Strain Reo/broiler/IN/A2323728-003/23: L1 (PX115322), L2 (PX115323), L3 (PX115324), M1 (PX115325), M2 (PX115326), M3 (PX115327), S1 (PX115328), S2 (PX115329), S3 (PX115330), S4 (PX115331). • Strain RVA/broiler/IN/A2323728-003/23/G19P[31]: VP1 (PX115332), VP2 (PX115333), VP3 (PX115334), VP4 (PX115335), NSP1 (PX115336), VP6 (PX115337), NSP3 (PX115338), NSP2 (PX115339), VP7 [Tx] (PX115340), NSP4 (PX115341), NSP5 (PX115342). Raw sequencing data are available in the NCBI Sequence Read Archive (SRA) under run accession number SRR34927529, associated with BioSample SAMN50525953 and BioProject PRJNA1142602.

## References

[B1] Álvarez-Narváez S. Harrell T. L. Nour I. Mohanty S. K. Conrad S. J. (2025). Choosing the most suitable NGS technology to combine with a standardized viral enrichment protocol for obtaining complete avian orthoreovirus genomes from metagenomic samples. Front. Bioinform. 5. doi: 10.3389/fbinf.2025.1498921, PMID: 39967836 PMC11833334

[B2] Ayalew L. E. Ahmed K. A. Popowich S. Lockerbie B.-C. Gupta A. Tikoo S. K. . (2022). Virulence of emerging arthrotropic avian reoviruses correlates with their ability to activate and traffic interferon-γ producing cytotoxic CD8+ T cells into gastrocnemius tendon. Front. Microbiol. 13. doi: 10.3389/fmicb.2022.869164, PMID: 35369435 PMC8964311

[B3] Benavente J. Martínez-Costas J. (2007). Avian reovirus: structure and biology. Virus Res. 123, 105–119. doi: 10.1016/j.virusres.2006.09.005, PMID: 17018239

[B4] Berg T. P. V. D. (2000). Acute infectious bursal disease in poultry: a review. Avian Pathol. 29, 175–194. doi: 10.1080/03079450050045431, PMID: 19184804

[B5] Beserra L. A. R. Barbosa B. R. P. Bernardes N. T. C. G. Brandão P. E. Gregori F. (2014). Occurrence and characterization of rotavirus A in broilers, layers, and broiler breeders from Brazilian poultry farms. Avian Dis. 58, 153–157. doi: 10.1637/10626-080513-ResNote.1, PMID: 24758129

[B6] Boros Á. Pankovics P. Adonyi Á. Fenyvesi H. Day J. M. Phan T. G. . (2016). A diarrheic chicken simultaneously co-infected with multiple picornaviruses: complete genome analysis of avian picornaviruses representing up to six genera. Virology 489, 63–74. doi: 10.1016/j.virol.2015.12.002, PMID: 26707271

[B7] Boros Á. Pankovics P. Knowles N. J. Nemes C. Delwart E. Reuter G. (2014). Comparative complete genome analysis of chicken and Turkey megriviruses (family *Picornaviridae*): long 3′ untranslated regions with a potential second open reading frame and evidence for possible recombination. J. Virol. 88, 6434–6443. doi: 10.1128/jvi.03807-13, PMID: 24672039 PMC4093843

[B8] Capella-Gutiérrez S. Silla-Martínez J. M. Gabaldón T. (2009). trimAl: a tool for automated alignment trimming in large-scale phylogenetic analyses. Bioinformatics 25, 1972–1973. doi: 10.1093/bioinformatics/btp348, PMID: 19505945 PMC2712344

[B9] Chen L. Chen L. Wang X. Huo S. Li Y. (2022). Detection and molecular characterization of enteric viruses in poultry flocks in Hebei province, China. Animals 12, 2873. doi: 10.3390/ani12202873, PMID: 36290263 PMC9598388

[B10] Chen S. Yang J. Li L. Guo Y. Yang S. Su Z. . (2025). Characterization and pathogenicity of a novel avian orthoreovirus in China. Front. Microbiol. 15. doi: 10.3389/fmicb.2024.1529351, PMID: 39850133 PMC11754254

[B11] Conceição-Neto N. Zeller M. Lefrère H. De Bruyn P. Beller L. Deboutte W. . (2015). Modular approach to customise sample preparation procedures for viral metagenomics: a reproducible protocol for virome analysis. Sci. Rep. 5, 16532. doi: 10.1038/srep16532, PMID: 26559140 PMC4642273

[B12] Devaney R. Trudgett J. Trudgett A. Meharg C. Smyth V. (2016). A metagenomic comparison of endemic viruses from broiler chickens with runting-stunting syndrome and from normal birds. Avian Pathol. 45, 616–629. doi: 10.1080/03079457.2016.1193123, PMID: 27215546 PMC7113909

[B13] Dhama K. Saminathan M. Karthik K. Tiwari R. Shabbir M. Z. Kumar N. . (2015). Avian rotavirus enteritis – an updated review. Vet. Q. 35, 142–158. doi: 10.1080/01652176.2015.1046014, PMID: 25917772

[B14] Fahey J. Crawley J. (1954). Studies on chronic respiratory disease of chickens II. Isolation of a virus. Can. J. Comp. Med. Vet. Sci. 18, 13–21. 17648682 PMC1791638

[B15] Falkenhagen A. Tausch S. H. Labutin A. Grützke J. Heckel G. Ulrich R. G. . (2022). Genetic and biological characteristics of species A rotaviruses detected in common shrews suggest a distinct evolutionary trajectory. Virus Evol. 8, veac004. doi: 10.1093/ve/veac004, PMID: 35169491 PMC8838746

[B16] Farkas T. Fey B. Hargitt E. Parcells M. Ladman B. Murgia M. . (2012). Molecular detection of novel picornaviruses in chickens and Turkeys. Virus Genes 44, 262–272. doi: 10.1007/s11262-011-0695-4, PMID: 22160827 PMC7089249

[B17] Gallego J. C. Lorencena D. de Mello J. L. Delai R. R. de Matos M. R. de Marco Viott A. . (2022). Investigation of avian rotavirus infections in broiler chicks from commercial flocks with different performance efficiency indexes. Vet. Res. Commun. 46, 853–858. doi: 10.1007/s11259-022-09910-x, PMID: 35229242 PMC8885118

[B18] Gerber P. F. Shen H. Zheng Y. Li G. Lobato Z. I. Opriessnig T. (2019). Genomic sequence of a *Megrivirus* strain identified in laying hens in Brazil. Microbiol. Resour. Announc. 8, e01438–e01418. doi: 10.1128/mra.01438-18, PMID: 30701237 PMC6346186

[B19] Grafl B. Gaußmann B. Bilic I. Folkertsma R. Hess M. (2025). Influence of biosecurity on the occurrence of various enteric viruses in broiler flocks. Avian Pathol. 54, 50–61. doi: 10.1080/03079457.2024.2377337, PMID: 39114873

[B20] Hoxie I. Dennehy J. J. (2020). Intragenic recombination influences rotavirus diversity and evolution. Virus Evol. 6, vez059. doi: 10.1093/ve/vez059, PMID: 31949920 PMC6955627

[B21] Hoxie I. Dennehy J. J. (2021). Rotavirus A genome segments show distinct segregation and codon usage patterns. Viruses 13, 1460. doi: 10.3390/v13081460, PMID: 34452326 PMC8402926

[B22] Jones D. T. Taylor W. R. Thornton J. M. (1992). The rapid generation of mutation data matrices from protein sequences. Comput. Appl. Biosci. 8, 275–282. doi: 10.1093/bioinformatics/8.3.275, PMID: 1633570

[B23] Kang K.-I. El-Gazzar M. Sellers H. S. Dorea F. Williams S. M. Kim T. . (2012). Investigation into the aetiology of runting and stunting syndrome in chickens. Avian Pathol. 41, 41–50. doi: 10.1080/03079457.2011.632402, PMID: 22845320

[B24] Kariithi H. M. Volkening J. D. Leyson C. M. Afonso C. L. Christy N. Decanini E. L. . (2022). Genome sequence variations of infectious bronchitis virus serotypes from commercial chickens in Mexico. Front. Vet. Sci. 9. doi: 10.3389/fvets.2022.931272, PMID: 35903135 PMC9315362

[B25] Katoh K. Standley D. M. (2013). MAFFT multiple sequence alignment software version 7: improvements in performance and usability. Mol. Biol. Evol. 30, 772–780. doi: 10.1093/molbev/mst010, PMID: 23329690 PMC3603318

[B26] Kohl C. Brinkmann A. Dabrowski P. W. Radonić A. Nitsche A. Kurth A. (2015). Protocol for metagenomic virus detection in clinical specimens. Emerg. Infect. Dis. 21, 48. doi: 10.3201/eid2101.140766, PMID: 25532973 PMC4285256

[B27] Kubacki J. Qi W. Fraefel C. (2022). Differential viral genome diversity of healthy and RSS-affected broiler flocks. Microorganisms 10, 1092. doi: 10.3390/microorganisms10061092, PMID: 35744610 PMC9231120

[B28] Kumar S. Stecher G. Li M. Knyaz C. Tamura K. (2018). MEGA X: molecular evolutionary genetics analysis across computing platforms. Mol. Biol. Evol. 35, 1547–1549. doi: 10.1093/molbev/msy096, PMID: 29722887 PMC5967553

[B29] Kunić V. Gottstein Ž. Prišlin M. Savić V. Brnić D. (2024). Health repercussions of avian rotaviruses on poultry and fancy pigeons. Vet. stn. 55, 677–689. doi: 10.46419/vs.55.6.3

[B30] Kwok K. T. de Rooij M. M. Messink A. B. Wouters I. M. Koopmans M. P. Phan M. V. (2020). Genome sequences of seven *Megrivirus* strains from chickens in the Netherlands. Microbiol. Resour. Announc. 9, e01207–e01220. doi: 10.1128/mra.01207-20, PMID: 33214312 PMC7679105

[B31] Kwok K. T. de Rooij M. M. Messink A. B. Wouters I. M. Smit L. A. Cotten M. . (2022). Establishing farm dust as a useful viral metagenomic surveillance matrix. Sci. Rep. 12, 16308. doi: 10.1038/s41598-022-20701-x, PMID: 36175536 PMC9521564

[B32] Li D. Liu C.-M. Luo R. Sadakane K. Lam T.-W. (2015). MEGAHIT: an ultra-fast single-node solution for large and complex metagenomics assembly via succinct *de Bruijn* graph. Bioinformatics 31, 1674–1676. doi: 10.1093/bioinformatics/btv033, PMID: 25609793

[B33] Lima D. A. Cibulski S. P. Tochetto C. Varela A. P. M. Finkler F. Teixeira T. F. . (2019). The intestinal virome of malabsorption syndrome-affected and unaffected broilers through shotgun metagenomics. Virus Res. 261, 9–20. doi: 10.1016/j.virusres.2018.12.005, PMID: 30543873

[B34] Liu H. J. Lee L. H. Hsu H. W. Kuo L. C. Liao M. H. (2003). Molecular evolution of avian reovirus: evidence for genetic diversity and reassortment of the S-class genome segments and multiple cocirculating lineages. Virology 314, 336–349. doi: 10.1016/S0042-6822(03)00415-X, PMID: 14517086

[B35] Liu L. Lu X. Guo X. Gong X. Hu F. Jiang Y. . (2025). Phylogenetic analysis and pathogenicity of avian reoviruses isolated from viral arthritis cases in China 2010–2024. Vet. Sci. 12, 307. doi: 10.3390/vetsci12040307, PMID: 40284810 PMC12031418

[B36] López S. Sánchez-Tacuba L. Moreno J. Arias C. F. (2016). Rotavirus strategies against the innate antiviral system. Annu. Rev. Virol. 3, 591–609. doi: 10.1146/annurev-virology-110615-042152, PMID: 27482897

[B37] Martin D. P. Murrell B. Golden M. Khoosal A. Muhire B. (2015). RDP4: Detection and analysis of recombination patterns in virus genomes. Virus Evol. 1, vev003. doi: 10.1093/ve/vev003, PMID: 27774277 PMC5014473

[B38] Matthijnssens J. Attoui H. Bányai K. Brussaard C. P. Danthi P. Del Vas M. . (2022). ICTV virus taxonomy profile: Spinareoviridae 2022. J. Gen. Virol. 103. doi: 10.1099/jgv.0.001781, PMID: 36394457 PMC12642366

[B39] Matthijnssens J. Ciarlet M. McDonald S. M. Attoui H. Bányai K. Brister J. R. . (2011). Uniformity of rotavirus strain nomenclature proposed by the Rotavirus Classification Working Group (RCWG). Arch. Virol. 156, 1397–1413. doi: 10.1007/s00705-011-1006-z, PMID: 21597953 PMC3398998

[B40] Matthijnssens J. Ciarlet M. Rahman M. Attoui H. Bányai K. Estes M. K. . (2008). Recommendations for the classification of group A rotaviruses using all 11 genomic RNA segments. Arch. Virol. 153, 1621–1629. doi: 10.1007/s00705-008-0155-1, PMID: 18604469 PMC2556306

[B41] McDonald S. M. Nelson M. I. Turner P. E. Patton J. T. (2016). Reassortment in segmented RNA viruses: mechanisms and outcomes. Nat. Rev. Microbiol. 14, 448–460. doi: 10.1038/nrmicro.2016.46, PMID: 27211789 PMC5119462

[B42] Müller H. Islam M. R. Raue R. (2003). Research on infectious bursal disease - the past, the present and the future. Vet. Microbiol. 97, 153–165. doi: 10.1016/j.vetmic.2003.08.005, PMID: 14637046

[B43] Nichols S. L. Haller C. Borodavka A. Esstman S. M. (2024). Rotavirus NSP2: a Master orchestrator of early viral particle assembly. Viruses 16, 814. doi: 10.3390/v16060814, PMID: 38932107 PMC11209291

[B44] Nour I. Alvarez-Narvaez S. Harrell T. L. Conrad S. J. Mohanty S. K. (2023). Whole genomic constellation of avian reovirus strains isolated from broilers with arthritis in North Carolina, USA. Viruses 15, 2191. doi: 10.3390/v15112191, PMID: 38005869 PMC10675200

[B45] Nour I. Mohanty S. K. (2024). Avian reovirus: from molecular biology to pathogenesis and control. Viruses 16, 1966. doi: 10.3390/v16121966, PMID: 39772272 PMC11728826

[B46] Nwokorogu V. C. Pillai S. San J. E. Pillay C. Nyaga M. M. Sabiu S. (2024). A metagenomic investigation of the faecal RNA virome structure of asymptomatic chickens obtained from a commercial farm in Durban, KwaZulu-Natal province, South Africa. BMC Genomics 25, 629. doi: 10.1186/s12864-024-10517-6, PMID: 38914944 PMC11194887

[B47] Paez-Espino D. Pavlopoulos G. A. Ivanova N. N. Kyrpides N. C. (2017). Nontargeted virus sequence discovery pipeline and virus clustering for metagenomic data. Nat. Protoc. 12, 1673–1682. doi: 10.1038/nprot.2017.063, PMID: 28749930

[B48] Palomino-Tapia V. Mitevski D. Inglis T. van der Meer F. Abdul-Careem M. F. (2018). Molecular characterization of emerging avian reovirus variants isolated from viral arthritis cases in Western Canada 2012–2017 based on partial sigma (σ) C gene. Virology 522, 138–146. doi: 10.1016/j.virol.2018.06.006, PMID: 30029013

[B49] Pantin-Jackwood M. J. Spackman E. Michael Day J. Rives D. (2007). Periodic monitoring of commercial Turkeys for enteric viruses indicates continuous presence of astrovirus and rotavirus on the farms. Avian Dis. 51, 674–680. doi: 10.1637/0005-2086(2007)51[674:PMOCTF]2.0.CO;2 17992925

[B50] Patzina-Mehling C. Falkenhagen A. Trojnar E. Gadicherla A. K. Johne R. (2020). Potential of avian and mammalian species A rotaviruses to reassort as explored by plasmid only-based reverse genetics. Virus Res. 286, 198027. doi: 10.1016/j.virusres.2020.198027, PMID: 32442596

[B51] Rafique S. Rashid F. Wei Y. Zeng T. Xie L. Xie Z. (2024). Avian orthoreoviruses: a systematic review of their distribution, dissemination patterns, and genotypic clustering. Viruses 16, 1056. doi: 10.3390/v16071056, PMID: 39066218 PMC11281703

[B52] SAN Group (2021). Quick Guide: NGS Sampling Protocol Using AniCard. Available online at: https://www.anicon.eu/card (Accessed June 30, 2025).

[B53] Savita S. Kusumakar A. Malik Y. Minakshi M. Prasad G. (2008). Detection and characterization of group A and D avian rotaviruses in India. Indian J. Biotechnol. 7, 554–556.

[B54] Schat K. A. Skinner M. A. (2014). “ Chapter 16 - avian immunosuppressive diseases and immunoevasion,” in Avian Immunology. Eds. Schat K. A. Kaspers B. Kaiser P. ( Academic Press, Oxfordshire, UK), 275–297. doi: 10.1016/B978-0-12-396965-1.00016-9, PMID:

[B55] Singh K. Mehta D. Dumka S. Chauhan A. S. Kumar S. (2023). Quasispecies nature of RNA viruses: lessons from the past. Vaccines 11, 308. doi: 10.3390/vaccines11020308, PMID: 36851186 PMC9963406

[B56] Song X. Hao Y. (2009). Adaptive evolution of rotavirus VP7 and NSP4 genes in different species. Comput. Biol. Chem. 33, 344–349. doi: 10.1016/j.compbiolchem.2009.07.008, PMID: 19665933

[B57] Suzuki Y. Yaeshiro M. Uehara D. (2024). Intra-segmental recombinations between avian and mammalian VP4 genotypes in *Rotavirus alphagastroenteritidis*. Gene Rep. 37, 102063. doi: 10.1016/j.genrep.2024.102063, PMID: 41735180

[B58] Tang Y. Lin L. Sebastian A. Lu H. (2016). Detection and characterization of two co-infection variant strains of avian orthoreovirus (ARV) in young layer chickens using next-generation sequencing (NGS). Sci. Rep. 6, 24519. doi: 10.1038/srep24519, PMID: 27089943 PMC4835796

[B59] Trojnar E. Sachsenröder J. Twardziok S. Reetz J. Otto P. H. Johne R. (2013). Identification of an avian group A rotavirus containing a novel VP4 gene with a close relationship to those of mammalian rotaviruses. J. Gen. Virol. 94, 136–142. doi: 10.1099/vir.0.047381-0, PMID: 23052396

[B60] Van Borm S. Steensels M. Mathijs E. Vandenbussche F. van den Berg T. Lambrecht B. (2021). Metagenomic sequencing determines complete infectious bronchitis virus (avian *Gammacoronavirus*) vaccine strain genomes and associated viromes in chicken clinical samples. Virus Genes 57, 529–540. doi: 10.1007/s11262-021-01872-7, PMID: 34626348 PMC8501334

[B61] Walker E. R. Friedman M. Olson N. (1972). Electron microscopic study of an avian reovirus that causes arthritis. J. Ultrastruct. Res. 41, 67–79. doi: 10.1016/S0022-5320(72)90039-1, PMID: 4627607

