# Supplementary material for: Co-detection and genomic characterization of avian rotavirus A, avian orthoreovirus, and chicken megrivirus-C using nontargeted metagenomic surveillance in Indian broiler chickens
Source: Front Cell Infect Microbiol. 2026 Feb 27;16:1690222. doi: 10.3389/fcimb.2026.1690222 (PMC12982373; doi:10.3389/fcimb.2026.1690222)
Supplement: Supplementary file 1 [file Table1.docx]

**Co-Detection and Genomic Characterization of Avian Rotavirus A, Avian Orthoreovirus, and Chicken Megrivirus-C Using Nontargeted Metagenomic Surveillance in Indian Broiler Chickens**

**Henry M. Kariithi ^1, *^, Jeremy D. Volkening ^2^, Sarah N. Mueni ^3^, Mohamed Helmy ^4^, Claudio L. Afonso ^2^,** **Pushparaj P. Chaudhari ^5^, and Eduardo L. Decanini ^4, *^**

^1^ Biotechnology Research Institute, Kenya Agricultural and Livestock Research Organization, PO Box 362-00902, Kikuyu, Kenya. [henry.kariithi@kalro.org](mailto:henry.kariithi@kalro.org); ORCiD: [0000-0002-9250-9108](https://orcid.org/my-orcid?orcid=0000-0002-9250-9108).

^2^ BASE_2_BIO, Oshkosh, WI 54904-8221, USA. [jeremy.volkening@base2bio.com](file:///D:\USDA-ARS%20Secondment\BIAH%20Project\aMPVs%20(BIAH%20FTA%20Samples)\Manuscript\jeremy.volkening@base2bio.com); ORCiD: [0000-0002-8892-7155](https://orcid.org/0000-0002-8892-7155) (J.D.V.), [claudio.afonso@base2bio.com](file:///D:\USDA-ARS%20Secondment\BIAH%20Project\aMPVs%20(BIAH%20FTA%20Samples)\Manuscript\claudio.afonso@base2bio.com); ORCiD: [0000-0001-5699-4743](https://orcid.org/0000-0001-5699-4743) (C.L.A.).

^3^ Department of Biochemistry, Jomo Kenyatta University of Agriculture and Technology, Juja Kwale Road, PO Box 62000-00200, Nairobi, Kenya; [sarah.mueni@jkuat.ac.ke](mailto:sarah.mueni@jkuat.ac.ke).

^4^ Boehringer Ingelheim Middle East and North Africa, PO Box 507066, Dubai, United Arab Emirates; [eduardo.lucio@boehringer-ingelheim.com](mailto:eduardo.lucio@boehringer-ingelheim.com) (E.L.D.), [mohamed.helmy@boehringer-ingelheim.com](mailto:mohamed.helmy@boehringer-ingelheim.com) (M.H.).

5 Boehringer Ingelheim India Pvt. Ltd., Mumbai, Vikhroli East, Maharashtra 400079, India; [pushparaj.chaudhari@boehringer-ingelheim.com](mailto:pushparaj.chaudhari@boehringer-ingelheim.com).

*** Correspondence**:

Henry Kariithi (+254-702-117653; [henry.kariithi@kalro.org](mailto:Henry.Kariithi@kalro.org)); Eduardo Decanini ([+971-4-4230-455](mailto:+971-4-4230-455); [eduardo.lucio@boehringer-ingelheim.com](mailto:eduardo.lucio@boehringer-ingelheim.com)).

## Supplementary Tables

**Supplementary Table S1**. Vaccination schedules and product details for the three commercial broiler farms (Farms A, B, and C) sampled in Kamrup Rural District, Assam, Northeast India, for the current study. Vaccines were administered at the hatchery (Day 1) and again at Day 12, following standard regional protocols. Product descriptions highlight the vaccine type, immunological target, and manufacturer where available. All vaccines were applied using standard hatchery or farm-level delivery practices.

| **Age (Days)** | **Farm A**  **(19-day-old flock)** | **Farms B & C**  **(33-day-old flocks)** | **Product Descriptions** |
| --- | --- | --- | --- |
| Day 1 | Vectored HVT-ND vaccine | Vectored HVT-ND vaccine | HVT-vectored recombinant vaccines expressing ND virus antigens for long-term protection. |
|  | Immune complex vaccine | Immune complex vaccine | Immune complex vaccine targeting IBD, designed for early-life protection |
|  | IBH killed vaccine (local) | IBH killed vaccine (local) | Inactivated vaccine targeting inclusion body hepatitis, commonly formulated by local producers |
| Day 12 | ND live vaccine | ND live vaccine | ND live vaccine: Live attenuated booster to reinforce hatchery-initiated ND immunity. |

**Abbreviations**: HVT = herpesvirus of turkeys; IBD = infectious bursal disease; IBH = inclusion body hepatitis; ND = Newcastle disease

**Supplementary Table S2**. Polymorphisms identified in the genome sequences of avian rotavirus A strain AvRV-A/broiler/IN/A2323728-003/23/G19P[31], avian orthoreovirus strain Reo/broiler/IN/A2323728-003/23, and chicken megrivirus type C strain ChMeV-C/broiler/IN/A2323728-003/23 detected in a pooled cloacal sample from asymptomatic Indian broilers. A total of 91 polymorphisms were detected across the three viruses, comprising 75 synonymous and 16 nonsynonymous variants. Specifically, AvRV-A had 7 polymorphisms (4 synonymous, 3 nonsynonymous), orthoreovirus had 71 (63 synonymous, 8 nonsynonymous), and ChMeV-C had 13 (8 synonymous, 5 nonsynonymous). Variants were filtered using thresholds of ≥10% variant frequency, ≥10× read depth, and ≤40% strand bias. The codon changes and resulting amino acid residues are shown for each variant position. The observed range of read coverage depth at variant positions was 19×–245×, and variant frequencies ranged from 10.0% to 40.1%.

| **Virus strain** | **Segment** | **Encoded protein^a^** | **Nucleotide position** | **Consensus** | | **Variant** | | **Read coverage depth at variant position^b^** | **Variant frequency^c^** | **Polymorphism type (coding effect)^d^** |
| --- | --- | --- | --- | --- | --- | --- | --- | --- | --- | --- |
|  |  |  |  | **Codon** | **aa residue** | **Codon** | **aa residue** |  |  |  |
| AvRV-A/broiler/IN/A2323728-003/23/G19P[31] | 1 | VP1 (RNA-dependent RNA polymerase) | 921 | TT**A** | Leu | TT**G** | Leu | 135× | 11.1% | synonymous |
|  | 2 | VP2 (inner capsid protein) | 1,091 | GA**G** | Glu | GA**A** | Glu | 245× | 16.3% | synonymous |
|  |  |  | 2,571 | **G**CC | Ala | **A**CC | Thr | 70× | 20.0% | nonsynonymous (A852T) |
|  | 3 | VP3 (RNA capping enzyme) | 631 | TC**G** | Ser | TC**A** | Ser | 34× | 26.5% | synonymous |
|  | 6 | VP6 (intermediate capsid protein) | 131 | CA**A** | Gln | CA**G** | Gln | 217× | 40.1% | synonymous |
|  | 7 | VP7 (outer capsid glycoprotein) | 394 | TC**A** | Ser | TC**C** | Ser | 148× | 28.4% | synonymous |
|  |  |  | 545 | **T**GG | Trp | **C**GG | Arg | 130× | 11.5% | nonsynonymous (W168R) |
|  | 8 | NSP2 (viroplasm-associated RNA-binding protein) | 155 | G**C**T | Ala | G**T**T | Val | 181× | 31.5% | nonsynonymous (A37V) |
| Reo/broiler/IN/A2323728-003/23 | L1 | λA (major core shell protein) | 753 | **G**AT | Asp | **C**AT | His | 160× | 10.0% | nonsynonymous (D245H) |
|  |  |  | 1,145 | CT**A** | Leu | CT**G** | Leu | 99× | 11.1% | synonymous |
|  |  |  | 1,181 | GC**T** | Ala | GC**G** | Ala | 76× | 11.8% | synonymous |
|  |  |  | 1,220 | GT**T** | Val | GT**C** | Val | 93× | 11.8% | synonymous |
|  |  |  | 1,268 | CG**G** | Arg | CG**A** | Arg | 94× | 11.7% | synonymous |
|  |  |  | 1,400 | GC**C** | Ala | GC**A** | Ala | 129× | 10.9% | synonymous |
|  |  |  | 1,460 | AT**T** | Ile | AT**C** | Ile | 129× | 13.2% | synonymous |
|  |  |  | 1,466 | GC**C** | Ala | GC**T** | Ala | 133× | 13.5% | synonymous |
|  |  |  | 1,478 | GC**T** | Ala | GC**C** | Ala | 135× | 13.3% | synonymous |
|  |  |  | 1,523 | GC**A** | Ala | GC**G** | Ala | 114× | 12.3% | synonymous |
|  |  |  | 2,108 | CA**C** | His | CA**T** | His | 146× | 11.0% | synonymous |
|  |  |  | 2,120 | AT**C** | Ile | AT**T** | Ile | 114× | 12.3% | synonymous |
|  |  |  | 2,240 | AC**T** | Thr | AC**C** | Thr | 152× | 11.8% | synonymous |
|  |  |  | 2,375 | GG**G** | Gly | GG**T** | Gly | 239× | 10.5% | synonymous |
|  |  |  | 2,648 | CG**T** | Arg | CG**C** | Arg | 171× | 14.6% | synonymous |
|  |  |  | 2,693 | GC**C** | Ala | GC**T** | Ala | 144× | 13.2% | synonymous |
|  |  |  | 3,119 | GC**G** | Ala | GC**A** | Ala | 110× | 10.0% | synonymous |
|  |  |  | 3,146 | TC**T** | Ser | TC**G** | Ser | 101× | 10.9% | synonymous |
|  |  |  | 3,434 | GA**C** | Asp | GA**T** | Asp | 102× | 15.7% | synonymous |
|  |  |  | 3,453 | **C**TG | Leu | **T**TG | Leu | 109× | 14.7% | synonymous |
|  |  |  | 3,485 | CC**C** | Pro | CC**T** | Pro | 139× | 11.5% | synonymous |
|  |  |  | 3,500 | TA**C** | Tyr | TA**T** | Tyr | 141× | 11.3% | synonymous |
|  |  |  | 3,531 | **A**CG | Thr | **T**CG | Ser | 146× | 11.0% | nonsynonymous (T1171S) |
|  | L2 | λB (RNA-dependent RNA polymerase) | 908 | **T**TA | Leu | **C**TA | Leu | 146× | 11.0% | synonymous |
|  |  |  | 2,620 | TT**C** | Phe | TT**T** | Phe | 191× | 12.0% | synonymous |
|  |  |  | 3,187 | TC**A** | Ser | TC**C** | Ser | 107× | 12.1% | synonymous |
|  |  |  | 3,223 | CC**C** | Pro | CC**T** | Pro | 180× | 10.0% | synonymous |
|  | L3 | λC (core turret protein) | 1,946 | C**T**G | Leu | C**C**G | Pro | 77× | 11.7% | nonsynonymous (L645P) |
|  | M1 | μA (minor core NTPase protein) | 1,517 | GC**C** | Ala | GC**T** | Ala | 65× | 10.8% | synonymous |
|  |  |  | 2,039 | AG**G** | Arg | AG**A** | Ala | 44× | 22.7% | synonymous |
|  |  |  | 2,152 | G**G**T | Gly | GAT | Asp | 19× | 26.3% | nonsynonymous (G713D) |
|  | M2 | μB (major outer capsid protein) | 1,529 | GG**T** | Gly | GG**C** | Gly | 123× | 11.4% | synonymous |
|  | M3 | μNS (viroplasm matrix protein) | 357 | AT**T** | Ile | AT**C** | Ile | 205× | 12.7% | synonymous |
|  |  |  | 396 | AA**G** | Lys | AA**A** | Lys | 146× | 13.7% | synonymous |
|  |  |  | 438 | TT**C** | Phe | TT**T** | Phe | 159× | 12.6% | synonymous |
|  |  |  | 648 | AT**G** | Met | AT**A** | Ile | 213× | 17.8% | nonsynonymous (M208I) |
|  |  |  | 804 | GC**C** | Ala | GC**T** | Ala | 167× | 10.2% | synonymous |
|  |  |  | 882 | GA**A** | Glu | GA**G** | Glu | 148× | 14.2% | synonymous |
|  |  |  | 946 | **T**TT | Phe | **C**TT | Leu | 144× | 20.8% | nonsynonymous (F308L) |
|  |  |  | 1,029 | GT**C** | Val | GT**T** | Val | 134× | 29.9% | synonymous |
|  | S2 | σA (RNA-binding core protein) | 369 | GC**A** | Ala | GC**T** | Ala | 71× | 11.3% | synonymous |
|  |  |  | 1,131 | CA**G** | Gln | CA**A** | Gln | 91× | 16.5% | synonymous |
|  |  |  | 1,140 | GC**C** | Ala | GC**A** | Ala | 88× | 17.0% | synonymous |
|  |  |  | 1,146 | GA**C** | Asp | GA**T** | Asp | 78× | 19.2% | synonymous |
|  |  |  | 1,155 | TT**G** | Leu | TT**A** | Leu | 76× | 22.4% | synonymous |
|  |  |  | 1,161 | AA**C** | Asn | AA**T** | Asn | 90× | 10.0% | synonymous |
|  |  |  | 1,185 | AG**G** | Arg | AG**A** | Arg | 105× | 12.4% | synonymous |
|  |  |  | 1,188 | AT**C** | Ile | AT**T** | Ile | 103× | 12.6% | synonymous |
|  |  |  | 1,191 | AA**A** | Lys | AA**G** | Lys | 105× | 12.4% | synonymous |
|  |  |  | 1,197 | TT**C** | Phe | TT**T** | Phe | 105× | 15.2% | synonymous |
|  |  |  | 1,206 | GG**T** | Gly | TT**C** | Gly | 101× | 15.8% | synonymous |
|  |  |  | 1,209 | GA**C** | Asp | GA**T** | Asp | 101× | 15.8% | synonymous |
|  |  |  | 1,227 | AC**T** | Thr | AC**C** | Thr | 95× | 16.8% | synonymous |
|  |  |  | 1,230 | GC**G** | Ala | GC**A** | Ala | 95× | 18.9% | synonymous |
|  |  |  | 1,233 | CA**A** | Gln | CA**G** | Gln | 94× | 17.0% | synonymous |
|  | S3 | σB (outer capsid antigenic protein) | 562 | **TCT** | Ser | **GCT** | Ala | 158× | 12.0% | nonsynonymous (S178A) |
|  |  |  | 585 | CT**C** | Leu | CT**T** | Leu | 178× | 10.1% | synonymous |
|  |  |  | 603 | TC**C** | Ser | CT**T** | Ser | 199× | 11.1% | synonymous |
|  |  |  | 616 | **C**CT | Pro | **T**CT | Ser | 193× | 11.4% | nonsynonymous (P196S) |
|  |  |  | 630 | TT**C** | Phe | TT**T** | Phe | 192× | 11.5% | synonymous |
|  |  |  | 642 | CA**G** | Gln | CA**A** | Gln | 197× | 14.2% | synonymous |
|  | S4 | σNS (nonstructural RNA-binding protein) | 464 | GC**T** | Ala | GC**C** | Ala | 169× | 10.1% | synonymous |
|  |  |  | 713 | GA**A** | Glu | GA**G** | Glu | 131× | 13.0% | synonymous |
|  |  |  | 923 | CG**C** | Arg | CG**T** | Arg | 50× | 16.0% | synonymous |
|  |  |  | 974 | AA**C** | Asn | AA**T** | Asn | 37× | 21.6% | synonymous |
|  |  |  | 989 | GC**T** | Ala | GC**C** | Ala | 42× | 19.0% | synonymous |
|  |  |  | 994-995 | A**AA** | Lys | A**GG** | Arg | 45× | 17.8% | nonsynonymous (K324R) |
|  |  |  | 1,001 | TC**T** | Ser | TC**C** | Ser | 45× | 17.8% | synonymous |
|  |  |  | 1,034 | CT**T** | Leu | CT**C** | Leu | 34× | 11.8% | synonymous |
|  |  |  | 1,095 | **T**TG | Leu | **C**TG | Leu | 30× | 10.0% | synonymous |
| ChMeV-C/broiler/IN/A2323728-003/23 | VP0 | VP0 (capsid precursor: VP4/VP2) | 1,196 | AA**C** | Asn | AA**T** | Asn | 55× | 16.40% | synonymous |
|  |  |  | 1,564 | G**T**A | Val | G**G**A | Gly | 63× | 11.10% | nonsynonymous (V294G) |
|  | VP1 | VP1 (major capsid protein) | 2,542 | C**G**T | Arg | C**C**T | Pro | 39× | 15.40% | nonsynonymous (R68P) |
|  |  |  | 2,933 | CT**G** | Leu | CT**A** | Leu | 42× | 14.30% | synonymous |
|  | 2A2 | 2A2 (nonstructural protein) | 3,935 | GG**C** | Gly | GG**T** | Gly | 100× | 13.00% | synonymous |
|  |  |  | 3,998 | GT**C** | Val | GT**T** | Val | 94× | 12.80% | synonymous |
|  | 2B | 2B (nonstructural protein) | 4,709 | GC**A** | Ala | GC**T** | Ala | 28× | 17.90% | synonymous |
|  |  |  | 4,991 | TC**T** | Ser | TC**C** | Ser | 48× | 10.40% | synonymous |
|  | 3A | 3A (membrane-associated protein) | 6,359 | AG**A** | Arg | AG**G** | Arg | 26× | 23.10% | synonymous |
|  |  |  | 6,437 | TG**T** | Cys | TG**C** | Cys | 21× | 14.30% | synonymous |
|  |  |  | 6,455 | CC**C** | Pro | CC**T** | Pro | 23× | 13.00% | synonymous |
|  |  |  | 6,635 | AC**T** | Thr | AC**C** | Thr | 22× | 18.20% | synonymous |
|  | 3D | 3D (RNA-dependent RNA polymerase) | 7,805 | AT**C** | Ile | AT**T** | Ile | 31× | 12.90% | synonymous |
|  |  |  | 7,896 | **A**CT | Thr | **T**CT | Ser | 60× | 16.70% | nonsynonymous (T140S) |
|  |  |  | 8,691 | **C**AT | His | **A**AT | Asn | 91× | 24.20% | nonsynonymous (H405N) |
|  | putative ORF2 | protein of unknown function | 8,967 | A**C**C | Thr | A**T**C | Ile | 63× | 12.70% | nonsynonymous (T23I) |
|  |  |  | 9,070 | AG**A** | Arg | AG**G** | Arg | 40× | 17.50% | synonymous |

**^a^**The descriptions of the encoded proteins and their functions are based on: (Matthijnssens et al., 2008; Trojnar et al., 2013) – for RVA; (Benavente and Martínez-Costas, 2007; Liu et al., 2025) – for ARV; and (Boros et al., 2014; Gerber et al., 2019) – for ChMeV.

**^b^Read coverage depth**: number of reads mapped at the variant position (range: 19×–245×).

**^c^ Variant frequency**: proportion of reads supporting the variant nucleotide at that position (range: 10.0%–40.1%).

**^d^ Polymorphism type**: classification based on amino acid change; synonymous = no change, nonsynonymous = amino-acid change.

## Supplementary Figures


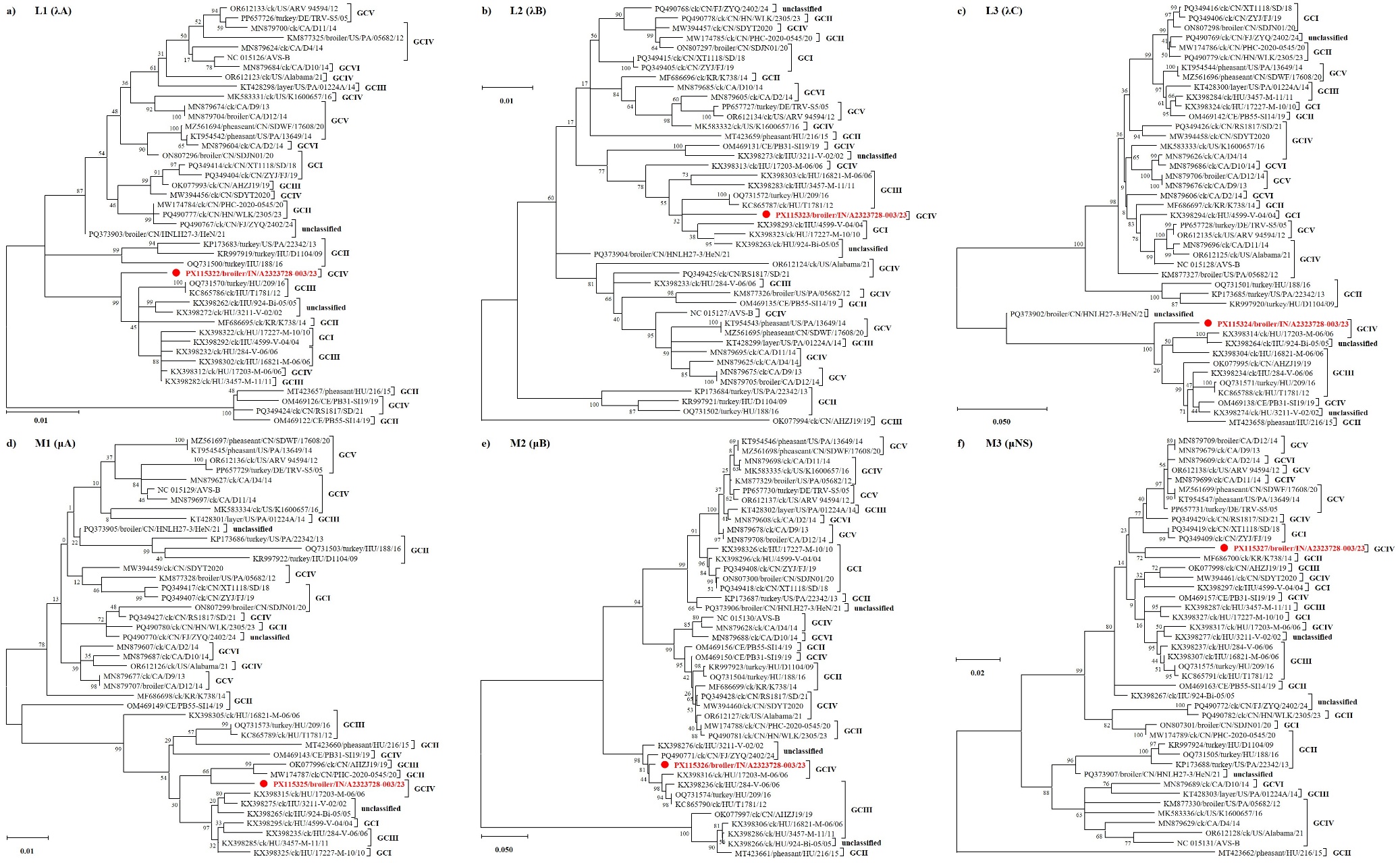


**Supplementary Figure S1**: Phylogenetic clustering of the Indian avian orthoreovirus strain Reo/broiler/IN/A2323728-003/23 identified in this study (highlighted in bold red) based on amino acid sequences of the coding regions (CDS) of the 10 ARV proteins encoded by genome segments L1 (λA), L2 (λB), L3 (λC), M1 (μA), M2 (μB), M3 (μNS), S1 (concatenated p10–p17–σC), S2 (σA), S3 (σB), and S4 (σNS). ML trees were constructed using the JTT matrix-based model with gamma-distributed rate variation and a proportion of invariant sites (JTT+G+I) (Jones et al., 1992). Each tree includes 43 sequences, with final alignment lengths of 1,293 (λA), 1,235 (λB), 1,277 (λC), 732 (μA), 675 (μB), 635 (μNS), 571 (S1), and 415 (σA), and 367 (σB and σNS) aa positions, respectively. Sequence labels indicate GenBank accession number, host species, country of origin (two-letter ISO code), strain name, and year of sample collection. Genotypic clusters (GCI–GCV, GCVI) are indicated by right square brackets on the trees.


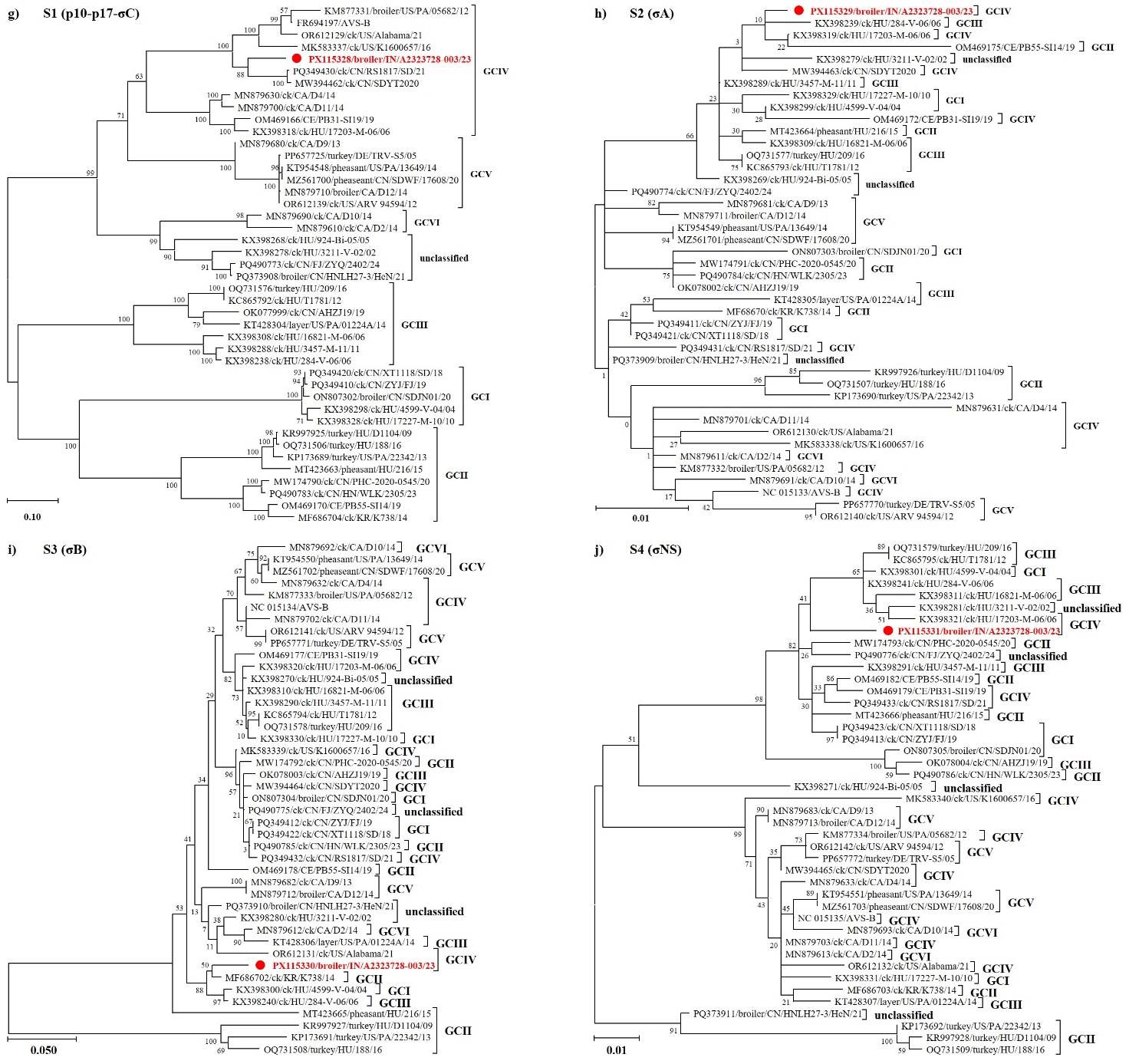


**Supplementary Figure S1 continued.**


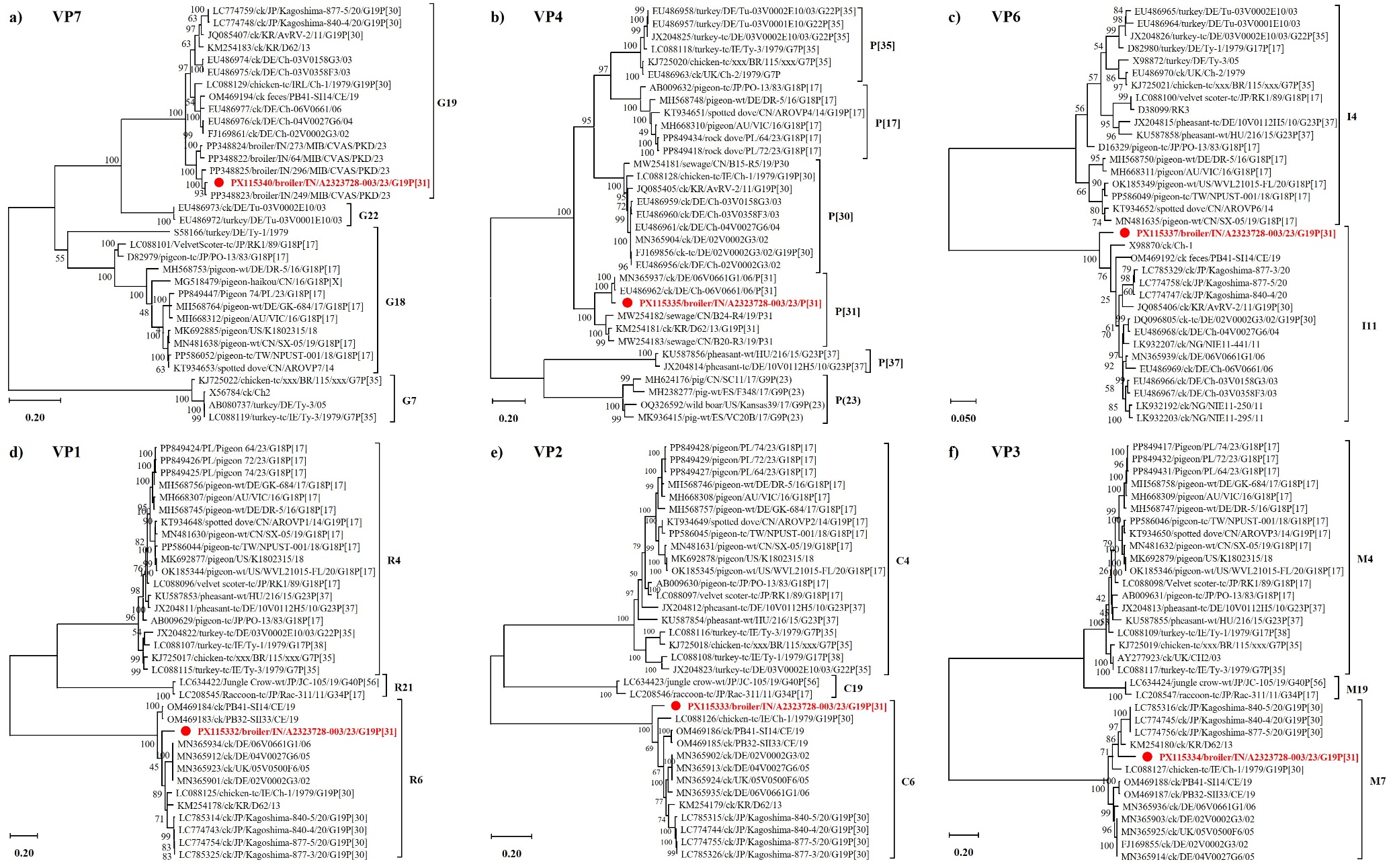


**Supplementary Figure S2**: Phylogenetic analysis of the Indian avian rotavirus A strain AvRV-A/broiler/IN/A2323728-003/23 identified in this study (highlighted in bold red), classified under the genotype constellation G19–P[31]–I11–R6–C6–M7–A16–N6–Tx–E10–H8. The ML trees were constructed by applying substitution models T92 (VP7, VP6, NSP2, NSP4, NSP5/6), HKY (VP4), TN93 (VP1, NSP3), and GTR (VP2, VP3, NSP1), each comprising 34 AvRV-A strains. Nucleotide alignment lengths were as follows: VP7 (905), VP4 (2,003), VP6 (1,194), VP1 (3,265), VP2 (2,667), VP3 (2,490), NSP1 (1,725), NSP2 (936), NSP3 (873), NSP4 (507), and NSP5/6 (627). Sequence labels indicate GenBank accession numbers, host species, country of origin (two-letter ISO code), strain name, and year of collection. Genotypic clusters are marked by right square brackets.


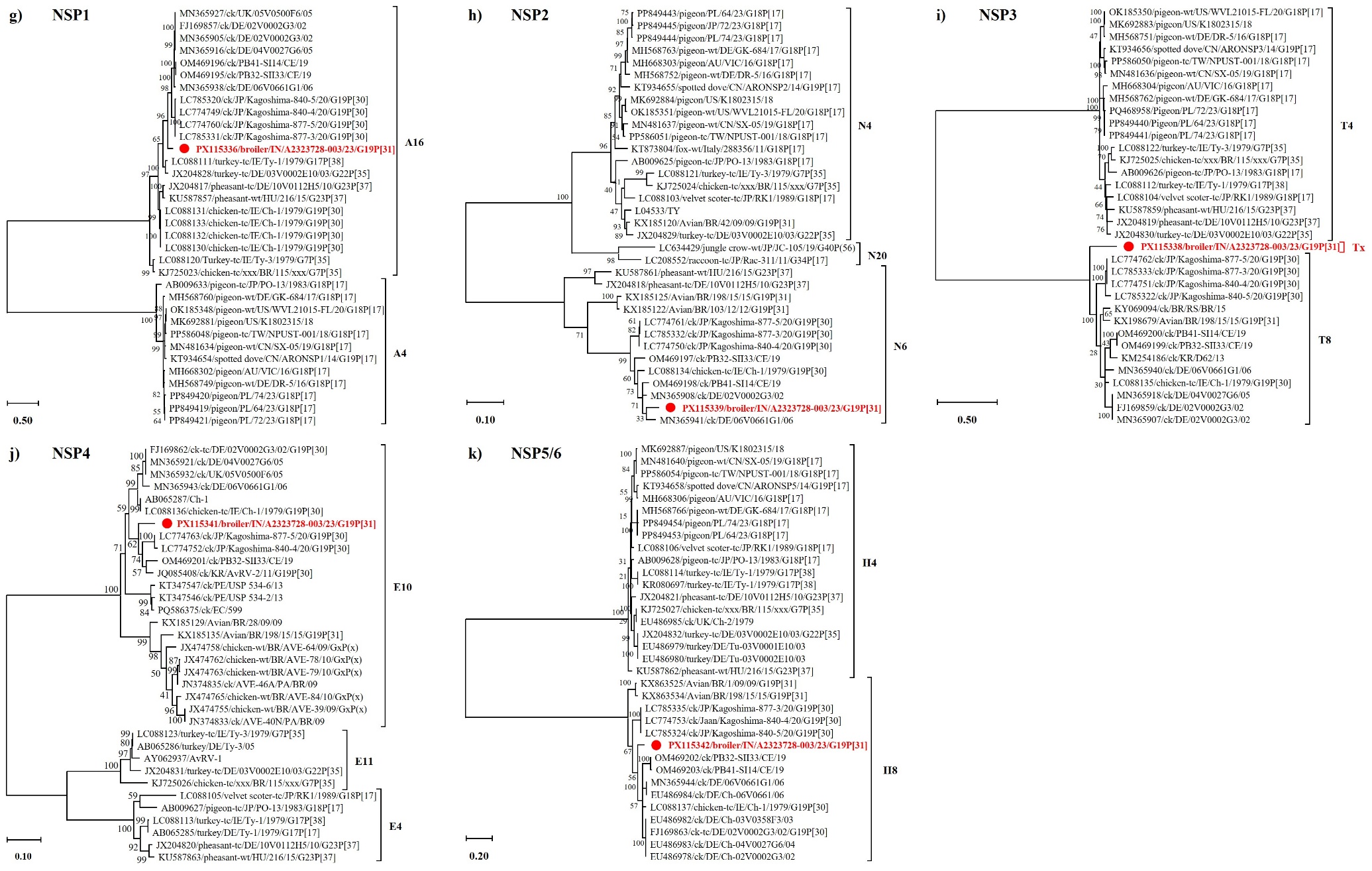


**Supplementary Figure S2 continued.**

# References

Benavente, J., and Martínez-Costas, J. (2007). Avian reovirus: structure and biology. *Virus Res.* 123, 105–119. doi: 10.1016/j.virusres.2006.09.005

Boros, Á., Pankovics, P., Knowles, N. J., Nemes, C., Delwart, E., and Reuter, G. (2014). Comparative complete genome analysis of chicken and turkey megriviruses (family *Picornaviridae*): long 3′ untranslated regions with a potential second open reading frame and evidence for possible recombination. *J. Virol.* 88, 6434–6443. doi: 10.1128/jvi.03807-13

Gerber, P. F., Shen, H., Zheng, Y., Li, G., Lobato, Z. I., and Opriessnig, T. (2019). Genomic sequence of a *Megrivirus* strain identified in laying hens in Brazil. *Microbiol. Resour. Announc.* 8, e01438-18. doi: 10.1128/mra.01438-18

Jones, D. T., Taylor, W. R., and Thornton, J. M. (1992). The rapid generation of mutation data matrices from protein sequences. *Comput. Appl. Biosci.* 8, 275–282. doi: 10.1093/bioinformatics/8.3.275

Liu, L., Lu, X., Guo, X., Gong, X., Hu, F., Jiang, Y., et al. (2025). Phylogenetic analysis and pathogenicity of avian reoviruses isolated from viral arthritis cases in China 2010–2024. *Vet. Sci.* 12, 307. doi: 10.3390/vetsci12040307

Matthijnssens, J., Ciarlet, M., Rahman, M., Attoui, H., Bányai, K., Estes, M. K., et al. (2008). Recommendations for the classification of group A rotaviruses using all 11 genomic RNA segments. *Arch. Virol.* 153, 1621–1629. doi: 10.1007/s00705-008-0155-1

Trojnar, E., Sachsenröder, J., Twardziok, S., Reetz, J., Otto, P. H., and Johne, R. (2013). Identification of an avian group A rotavirus containing a novel VP4 gene with a close relationship to those of mammalian rotaviruses. *J. Gen. Virol.* 94, 136–142. doi: 10.1099/vir.0.047381-0
